# Synaptobrevin-2 disease variants reveal spatial constraints within the presynaptic active zone

**DOI:** 10.1073/pnas.2507347122

**Published:** 2025-10-30

**Authors:** Natalie J. Guzikowski, Elena D. Bagatelas, Ok-Ho Shin, Yousuf A. Khan, Luis Esquivies, Baris Alten, Axel T. Brunger, Ege T. Kavalali

**Affiliations:** ^a^Department of Pharmacology, Vanderbilt University, Nashville, TN 37240-7933; ^b^Vanderbilt Brain Institute, Vanderbilt University, Nashville, TN 37240-7933; ^c^Department of Molecular and Cellular Physiology, Stanford University, Stanford, CA 94305; ^d^Department of Neurology and Neurological Sciences, Stanford University, Stanford, CA 94305; ^e^Department of Structural Biology, Stanford University, Stanford, CA 94305; ^f^Department of Photon Science, Stanford University, Stanford, CA 94305; ^g^HHMI, Stanford University, Stanford, CA 94305

**Keywords:** synaptobrevin-2, active zone, spontaneous neurotransmission

## Abstract

Soluble N-ethylmaleimide-sensitive factor attachment protein receptor (SNARE) proteins drive synaptic transmission in a temporally and spatially precise manner. Recent studies have identified several disease-causing SNARE variants that give rise to developmental and epileptic encephalopathies, defined as SNAREopathies. Here, we investigated nine synaptobrevin-2 (VAMP2) disease-causing variants and uncovered their specific SNARE complex affinity, stability, and conformational deficits that drive dysregulated neurotransmission. The neurotransmission deficits we observed parallel the symptomatic heterogeneity of the patients, with some variants displaying a disproportionate augmentation of spontaneous neurotransmitter release. When we examined the spatial organization of this excessive spontaneous release at nanoscale, we found that SNARE complexes composed of these variants formed exclusively outside of RIM scaffolding, revealing a preserved exclusion zone sparing evoked release from pathophysiology. Taken together with the phenotypes of previously reported disease-causing SNARE variants, these findings reveal shared patterns of aberrant neurotransmission across different SNAREs, highlighting the necessity for a functional classification of SNAREopathies to develop therapeutic interventions. The use of clinically relevant genetic manipulations to challenge the synapse provides mechanistic insight into rare diseases while simultaneously revealing fundamental aspects of synaptic physiology.

## Significance

The investigation into the pathophysiology of rare de novo variants in the integral soluble N-ethylmaleimide-sensitive factor attachment protein receptor (SNARE) complex protein, synaptobrevin-2, reveals fundamental principles of synaptic physiology. The nine synaptobrevin-2 disease-causing variants each uniquely disrupt SNARE complex formation and subsequent neurotransmission, thus revealing distinct synaptic phenotypes. These clinically relevant genetic manipulations illuminate the dynamic relationship between SNARE mediated release machinery and the structure of the synapse, where RIM liquid–liquid phase separation is integral in maintaining the fundamental segregation of different modes of release within the single micron of the synapse.

Developmental and epileptic encephalopathies (DEEs) are a group of disorders characterized by early-onset often intractable seizures along with significant developmental delay and intellectual disability. SNAREopathies have recently been described as a functional subclass of DEEs due to mutations in the SNARE complex or associated auxiliary proteins (1). Since DEEs tend to present similarly in infancy and early childhood with seizures and developmental delay, the diagnosis relies on identifying the culprit mutation rather than on clinical presentation alone. Synaptobrevin-2 (syb2 or VAMP2) is one of the key SNARE proteins essential for normal synaptic function, however, only recently, due to whole exome/genome sequencing, has the occurrence of Syb2 mutations been recognized (2–4), highlighting the gap in our understanding of Syb2-associated SNAREopathies. While Syb2’s role at the synapse is undisputedly critical for exocytosis and endocytosis, it remains unknown how individual de novo mutations alter neurotransmitter release and subsequently, cause a variable synaptic and clinical phenotype (5, 6). These rare Syb2 SNAREopathies afford the opportunity to unravel the intricacies of presynaptic function, as they have a clear genetic substrate for disease with documented clinical phenotypes but lack molecular and functional insight. The mechanistic investigation conducted here illuminates the relationship between the SNARE mediated release machinery and the structure of the synapse in dually regulating basal neurotransmission.

The evolutionary conserved SNARE proteins form the core membrane fusion machinery vital for neurotransmission. At the pre-synapse, the formation of the 4 helical SNARE bundle to execute synaptic vesicle fusion involves the vesicular SNARE, Syb2, and two target SNAREs, Syntaxin-1 and Synaptosomal-Associated Protein 25 kDa (SNAP25) (7). Interactions between these 3 SNARE proteins are specific and discrete, making Syb2 essential for vesicle exocytosis in both action potential evoked and spontaneous neurotransmission (5, 8). Multiple other proteins interact with the SNARE complex and are fundamental in executing release, including the late priming and regulatory protein complexin (9, 10), Ca^2+^ sensing protein Synaptotagmin-1 (11), trans-SNARE stimulatory protein Munc-18 (12, 13), priming factor Munc13 (14), and scaffolding protein RIM (15, 16), all associated with their own respective SNAREopathies (1). How the integrity of each Syb2 variant SNARE complex operates within this pre-synaptic protein matrix and how this, in turn, modulates the fidelity of neurotransmission will be the focus of this investigation.

In 2020, there were eleven identified patients with de novo non-synonymous Syb2 disease-causing variants (17–19). Nine of these variants are the focus of our study, as they span the SNARE motif with mutations in both the C- and N-terminal domains (17–19). All variants are de novo and heterozygous with distinct clinical features, including moderate-to-severe intellectual disability, delays in various cognitive domains, various seizure frequency and semiology, and additional neurological and neuropsychiatric features as presented in Table 1. The recent evaluation of SNAP25 SNAREopathies revealed how single mutations in SNAP25 result in distinct neurotransmission abnormalities correlated with the phenotypes of their respective clinical features (3), suggesting the functional grouping of these variants is far more clinically relevant than grouping by individual affected SNARE proteins. We used Syb2 variants to investigate the convergence and divergence of synaptic disease mechanisms to explore whether a similar functional grouping is applicable, which ultimately can lead to the development of targeted treatments for SNAREopathies based on various specific synaptic phenotypes (3, 18, 20).

**Table 1.** Clinical features of Syb2 SNAREopathy patients

Variant	p. Val43del (V43del) c. 128_130delTGG de novo	p. Ile45del (I45del) c. 135_137delCAT de novo	p. Arg56Leu (R56L) c. 167G>T de novo	p. Arg56X (R56X) c. 166C>T de novo	p. Ala67Pro (A67P) c. 199C>G de novo	p. Gly73Trp (G73W) c. 217G>T de novo	p. Ser75Pro (S75P) c. 223T>C de novo	p. Phe77Ser (F77S) c. 230T>C de novo	p. Glu78Ala (E78A) c. 233A>C de novo
Sex and age	M/14 y	F/3 y	M/5 y	F/20 y	M/13 y	M/39 y	F/3 y	M/13 y	M/10 y
Clinical seizure type	Focal seizures	None	Infantile spasms, focal, and tonic seizures	None	West syndrome, myoclonic seizures	Generalized convulsions	None	West syndrome (infantile spasms, convulsive status epilepticus)	Focal seizures, generalized tonic–clonic seizures
Intellectual disability	Moderate	Moderate	Severe	ND	Severe	ND	Severe	Severe	Severe
ASD and like features	Yes, stereotyped hand movements (wringing), absent purposeful hand movements	Yes, stereotyped hand movements (washing)	Unable to assess due to severe ID	Autistic features	Absent purposeful hand movements	Autistic features	Yes, stereotyped hand movements, absent purposeful hand movements	Yes, stereotyped hand movements, absent purposeful hand movements	Yes, body rocking, head banging, screaming, absent purposeful hand movements
Vision	Visual fixation poor	Visual fixation poor	Cortical visual impairment	Visual acuity deficits	Visual fixation poor	Retinitis pigmentosa	Visual fixation poor	Visual fixation poor	Visual fixation poor
Movement disorder	No	No	No	Catatonia	Hyperkinetic movement	Nystagmus, progressive ataxia, tremor	Choreic movement, flapping, dystonic postures	Choreic movement, myoclonic jerks	Generalized chorea
Hypotonia	Yes	Yes	ND	ND	Yes	ND	Yes	Yes	Yes
Speech impairment	Only 5 to 10 words	Only 5 words	Absent speech	No	Absent speech	Echolalia	Absent speech	Absent speech	Absent speech
Additional features	Clumsiness, abnormal behavior	Abnormal behavior	Unable to assess due to severe ID	Hallucinations, delusions, anxiety, depression, aggressive outbursts, self-injurious behavior	Not described	Obsessive compulsive tendencies	Inability to walk	Abnormal behavior, inability to walk, severe constipation	Abnormal behavior, self-injury, inability to walk

p., designates location in the protein; c., designates location in the cDNA. ND stands for clinical feature not described. For further information on clinical features, refer to original publications with first reports (17–19).

Here, we dissected how nine Syb2 patient variants dysregulate evoked and spontaneous neurotransmission, thus revealing distinct synaptic phenotypes. We observed two key findings: First, each of the nine Syb2 variants exhibits a distinct neurotransmission deficit; and second, when examining specific variants in detail, we found that evoked and spontaneous release are affected differently with the same variant (i.e., both are not uniformly augmented). These release abnormalities are driven by alterations in SNARE complex affinity and subsequent formation. However, select variants do not disrupt neurotransmission equally across all modes of release, but disproportionately augment spontaneous neurotransmission, suggesting that SNARE machinery interactions alone are insufficient to fully explain the neurotransmission deficit. Visualization of SNARE complexes at the pre-synapse revealed that the active zone functions as an exclusion zone via nano-scale liquid–liquid phase separated boundaries, thereby compartmentalizing and regulating evoked release separably from spontaneous release. Consequently, each Syb2 variant must operate within the structural confines of the pre-synapse, ultimately shaping its synaptic phenotype. This suggests an equal correspondence between SNAREs and structural proteins in regulating release, highlighting the multifaceted nature of a single quantal release event.

## Results

### Syb2 Haploinsufficiency Has Minor Effects on Neurotransmission.

**Fig. 1.**
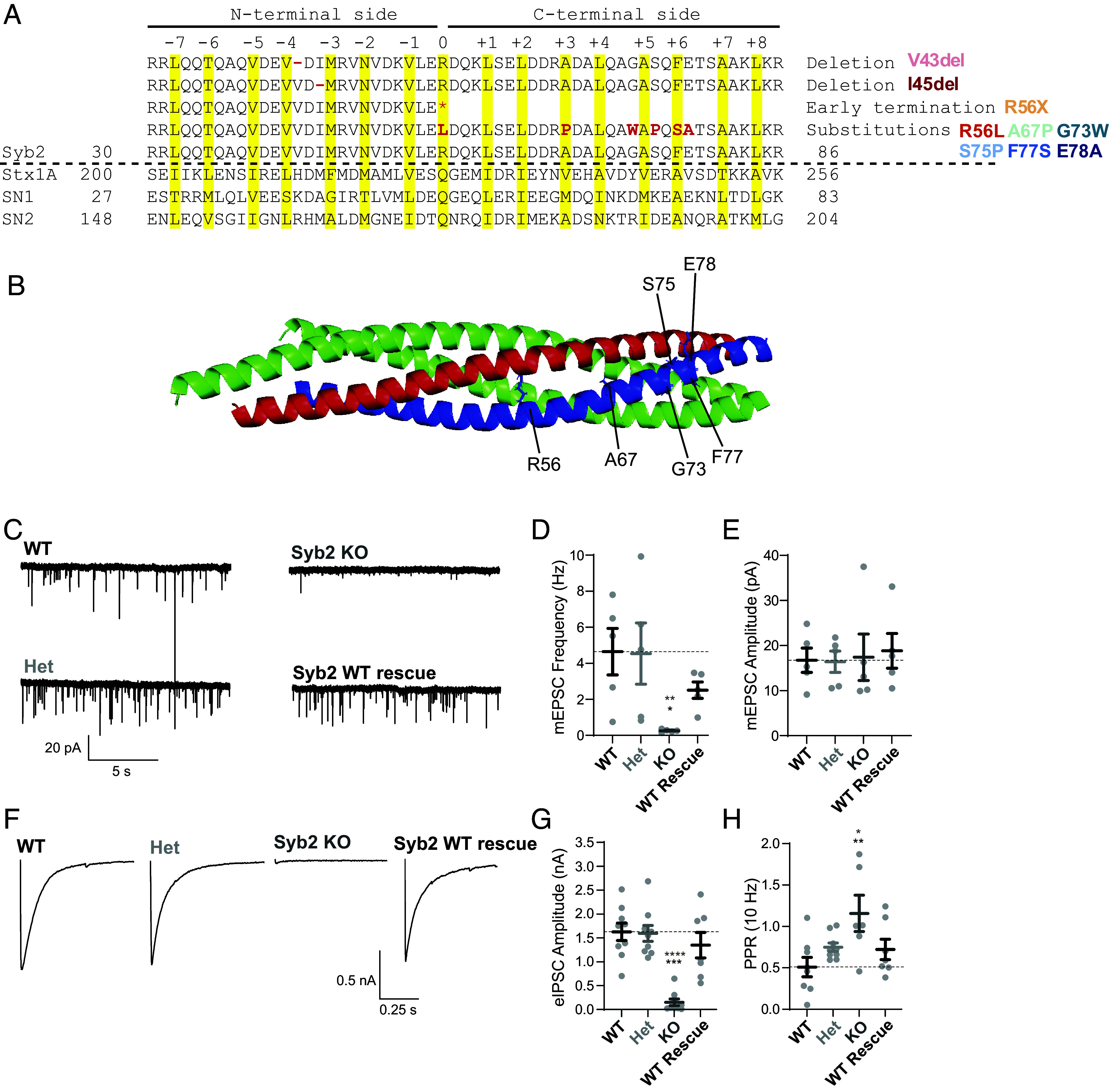
Syb2 haploinsufficiency has no significant effects on neurotransmission. (*A*) Amino acid sequence map of ternary SNARE complex with Syb2 patient variant mutations denoted in red (* indicating pre-mature truncation). SNARE motif hydrophobic layers and ionic layer zero are highlighted in yellow. (*B*) Structure of the SNARE complex with synaptobrevin-2 (syb2 blue), syntaxin-1a (syx1a RED), and SNAP25 (green). Substitution variants are denoted. (*C*) mEPSC example traces comparing genotypes of wildtype (Syb2^+/+^), heterozygous (Syb2^+/−^), Syb2 knock-out (Syb2^−/−^), and Syb2 knock-out with WT Syb2 rescue. (*D*) Quantification of mEPSC frequency and (*E*) amplitude between genotypes. (*F*) eIPSC example traces comparing genotypes of wildtype (Syb2^+/+^), heterozygous (Syb2^+/−^), Syb2 knock-out (Syb2^−/−^), and Syb2 knock-out with WT Syb2 rescue. (*G*) Quantification of eIPSC amplitude and (*H*) paired-pulse ratio between genotypes. Values are mean ± SEM. Significance reported as *P < 0.05, **P < 0.01, ***P < 0.001, and ****P < 0.0001 in black for multiple comparisons analysis and in brown for pairwise comparisons between each variant and WT genotype. Exact p-values, n numbers, and additional statistical information are provided in Dataset S2.

The SNARE complex is a parallel α-helical bundle with four individual SNARE motifs composed of Syb2, Syntaxin1, and SNAP25, which contributes not 1 but 2 SNARE motifs to complex formation. The interactions between the SNARE motifs form 15 hydrophobic zipper layers and one central ionic layer (21). The attachment of synaptic vesicles with the plasma membranes is driven by the zippering of the four helical bundle from the N-terminal to C-terminal side in the direction of the membrane (8, 22). Among the Syb2 variants studied, two mutations are located within the zipper layers (C-terminal side), two within the ionic layer (grouped with N-terminal variants), both deletion mutations are within the N-terminal domain and three mutations are located within the C-terminal domain (Fig. 1 *A*-*B*) (17–19).

All patients are heterozygous with the loss of one wild-type Syb2 allele, therefore, to examine if Syb2 SNAREopathy is due to a simple loss-of-function, we investigated synaptic transmission deficits in heterozygous (Syb2^+/−^) neurons relative to both knockout (Syb2^−/−^) and wildtype (Syb2^+/+^) genetic and rescue controls. As expected, Syb2^−/−^ neurons had a significant decrease in both mEPSC frequency and the magnitude of evoked release (5, 6). However, there were no significant alterations in spontaneous or evoked neurotransmission in Syb2^+/−^ neurons (Fig. 1 *C*–*H*). This is consistent with Syb2^+/−^ mice having relatively mild to absent behavioral phenotypes compared to littermate controls, with normal anxiety-like behaviors, fear conditioning, and startle responses (23). Thus, haploinsufficiency is not the sole driver of pathophysiology, but instead, the interference of variant Syb2 with wild-type Syb2 in a dominant manner may cause disease.

### Syb2 Variant Specific Alterations in Synaptic Transmission.

To elucidate how Syb2 variants impact neurotransmission, we overexpressed Syb2 variants on a wildtype background, in so doing mimicking the disease as patients have both wildtype and mutated Syb2 alleles. We first examined the electrophysiological effect of WT Syb2 overexpression at the synapse. Despite a ~threefold increase in WT Syb2 protein relative to empty vector control (*SI Appendix*, Fig. S1 *A* and *B*), we observe no significant effect on evoked inhibitory post-synaptic currents (eIPSCs) or evoked excitatory post-synaptic currents (eEPSCs) (*SI Appendix*, Fig. S2 *A*–*J*). Furthermore, when patient variants Syb2 are expressed, protein levels are comparable to WT (*SI Appendix*, Fig. S1 *A* and *B*, truncation mutation, R56X, is not recognized by the Syb2 antibody).

**Fig. 2.**
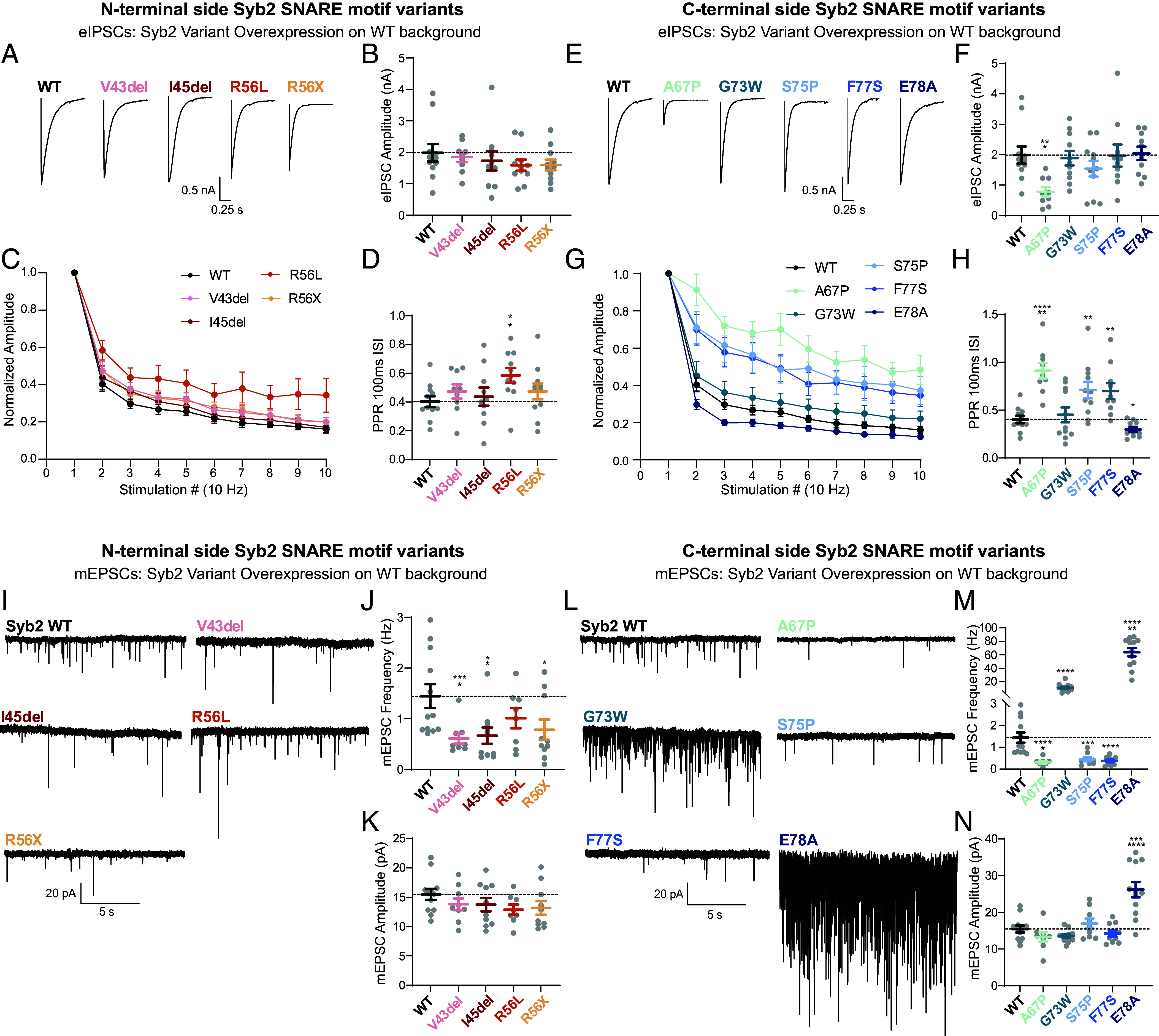
Syb2 variant specific alterations on synaptic transmission in the presence of wildtype Syb2. (*A*) Representative traces of WT Syb2 and N-terminal side Syb2 SNARE motif variants (including layer 0 variants) overexpressed in a WT genetic background. (*B*) Quantification of eIPSC amplitude in response to one stimulation and (*C*), repetitive stimulation (10 Hz), (*D*) with the first two responses used to calculate paired pulse ratio. (*E*) Representative traces of WT Syb2 and C-terminal side Syb2 SNARE motif variants overexpressed in a WT genetic background. (*F*) Quantification of eIPSC amplitude in response to one stimulation and (*G*) repetitive stimulation (10 Hz), (*H*) with the first two responses used to calculate paired pulse ratio. (*I*) mEPSC example traces of WT Syb2 and N-terminal side Syb2 SNARE motif variants (including layer 0 variants) overexpressed in a WT genetic background. (*J*) Quantification of mEPSC frequency and (*K*) amplitude. (*L*) mEPSC example traces of WT Syb2 and C-terminal side Syb2 SNARE motif variants overexpressed in a WT genetic background. (*M*) Quantification of mEPSC frequency and (*N*) amplitude. WT data is the same for N-terminal and C-terminal graphs. Values are mean ± SEM. Significance reported as *P < 0.05, **P < 0.01, ***P < 0.001, and ****P < 0.0001 in black for multiple comparisons analysis and in brown for pairwise comparisons between each variant and WT. Exact *P*-values, n numbers, and additional statistical information are provided in Dataset S2.

Due to network and recurrent activity, the direct impact of each Syb2 mutation on vesicle exocytosis is difficult to decipher in excitatory evoked recordings; therefore, the evaluation of eIPSCs will be the main source of quantitative comparison. Expression of N-terminal side Syb2 variants had no significant effect on evoked release (with the exception of R56L), with no change in eIPSC or eEPSC amplitudes. Only R56L had a decreased rate of eIPSC depression and a corresponding decrease in release probability (Fig. 2 *A*–*D*) (*SI Appendix*, Fig. S3 *A*–*D*). With C-terminal variant overexpression, A67P decreased eIPSC amplitude while A67P, S75P, and F77S all had a decreased rate of depression with repetitive stimulation and a corresponding decrease in release probability, similar to eEPSCs (Fig. 2 *E*–*H* and *SI Appendix*, Fig. S3 *E*–*H*). We hypothesize the variant A67P had the most robust effect on evoked release due to the introduction of a proline in a SNARE zipper layer, often regarded as the most disruptive amino acid substitution (24). Here, the G73W variant had a marginal effect on the rate of depression, differing from a previous report which documents a more pronounced effect on eIPSCs (18). Interestingly, the overexpression of only five of the nine Syb2 variants on a WT background resulted in significant deficits in evoked release, suggesting altered action potential dependent release does not ubiquitously underlie disease.

Next, we probed spontaneous action potential independent fusion of synaptic vesicles in the presence of tetrodotoxin (TTX) (25). We assessed the impact of patient variants on spontaneous miniature excitatory post-synaptic currents (mEPSCs) in a WT Syb2 background. Compared to WT overexpression (to account for protein levels potentially driving any electrophysiological phenotypes) (*SI Appendix*, Fig. S4 *A*–*F*), N-terminal variants V43del, I45del, and R56X caused a substantial decrease in mEPSC frequency, but not R56L (which had an altered evoked release probability) (Fig. 2 *I*–*K*). The C-terminal variants that had decreased evoked release probabilities (A67P, S75P, and F77S) also caused a decrease in mEPSC frequency [S75P consistent with liposome fusion assays (19)]. Conversely, G73W and E78A caused a robust increase in mEPSC frequency (ninefold and 44-fold, respectively, Fig. 2 *L*–*N*). Interestingly, Syb2 variant overexpression had a more substantial effect on mIPSC amplitude (*SI Appendix*, Fig. S3 *I*–*N*), suggesting a potential post-synaptic effect in receptor trafficking (26, 27). The synaptic transmission phenotypes observed in these overexpression experiments suggest a complex relationship between endogenous and variant Syb2 protein, potentially involving an interplay between Syb2 homo and hetero-dimers (28–30). Therefore, to clearly elucidate each variant’s ability to execute synaptic vesicle exocytosis, we next investigated evoked and spontaneous release in a Syb2^−/−^ background.

### Majority of Syb2 Variants Do Not Rescue Release in Syb2^−/−^ Background.

**Fig. 3.**
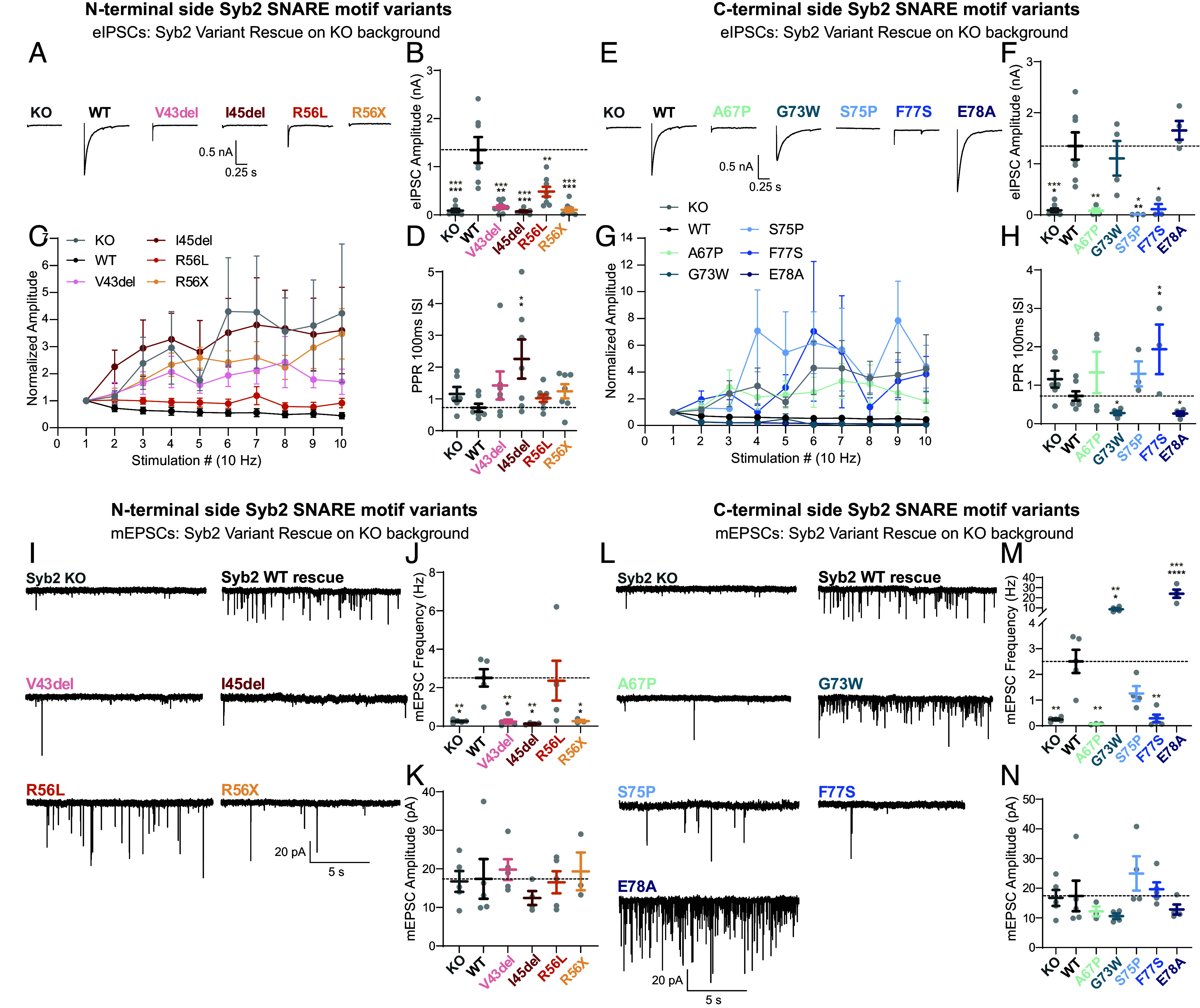
Syb2 patient variants bidirectionally dysregulate neurotransmission. (*A*) Representative traces of WT Syb2 and N-terminal side Syb2 SNARE motif variants (including layer 0 variants) expressed in a Syb2^−/−^ genetic background. (*B*) Quantification of eIPSC amplitude in response to one stimulation and (*C*) repetitive stimulation (10 Hz), (*D*) with the first two responses used to calculate paired pulse ratio. (*E*) Representative traces of WT Syb2 and C-terminal side Syb2 SNARE motif variants expressed in a Syb2^−/−^ genetic background. (*F*) Quantification of eIPSC amplitude in response to one stimulation and (*G*) repetitive stimulation (10 Hz), (*H*) with the first two responses used to calculate paired pulse ratio. (*I*) mEPSC example traces of WT Syb2 and N-terminal side Syb2 SNARE motif variants (including layer 0 variants) expressed in a Syb2^−/−^ genetic background. (*J*) Quantification of mEPSC frequency and (*K*) amplitude. (*L*) mEPSC example traces of WT Syb2 and C-terminal side Syb2 SNARE motif variants expressed in a Syb2^−/−^ genetic background. (*M*) Quantification of mEPSC frequency and (*N*) amplitude. KO and WT data are the same for N-terminal and C-terminal graphs. KO and WT rescue data are the same as for Fig. 1. Values are mean ± SEM. Significance reported as *P < 0.05, **P < 0.01, ***P < 0.001, and ****P < 0.0001 in black for multiple comparison analysis and in brown for pairwise comparisons between each variant and WT. Exact *P*-values, n numbers, and additional statistical information are provided in Dataset S2.

To ascertain whether the patient variants are sufficient to drive release in the absence of WT Syb2, we investigated synaptic transmission on a Syb2^−/−^ background. For the majority of C-terminal and N-terminal variants (7/9), we observe the variant Syb2 is not sufficient to rescue neurotransmission. No N-terminal variant was able to rescue evoked release (Fig. 3 *A* and *B*). Furthermore, each variant shows a shift to a more facilitatory phenotype, consistent with the Syb2^−/−^ electrophysiological phenotype (Fig. 3 *C* and *D*). C-terminal variants A67P, S75P, and F77S were also unable to rescue evoked release, and show a shift to a more facilitatory phenotype upon repetitive stimulation. However, C-terminal variants G73W and E78A both were sufficient to rescue eIPSC amplitudes and have an increased release probability (Fig. 3 *E*–*H*).

In line with evoked release, N-terminal variants V43del, I45del, and R56X were not able to rescue excitatory spontaneous release. Interestingly, R56L shows a comparable mEPSC frequency to WT rescue, consistent with results observed in R56L overexpression mEPSCs (Fig. 3 *I*–*K*). The comparison of N-terminal variants in the wildtype and knockout backgrounds suggests the effects of V43del and I45del on spontaneous release drive a dominant negative pathophysiology with R56X potentially causing haploinsufficiency. C-terminal variants A67P, S75P, and F77S were also unable to rescue mEPSCs, while S75P showed a milder phenotype with a less robust decrease in mEPSC frequency. Both G73W and E78A did not only rescue spontaneous release but had an aberrantly elevated spontaneous release rate (Fig. 3 *L*–*N*). While the changes observed in spontaneous release were robust, it is also plausible that even slight alterations in evoked release may underlie a component of disease. Taken together, the majority of Syb2 variants are insufficient to drive neurotransmission, but G73W and E78A uniquely display a consistent dominant positive phenotype across excitatory and inhibitory overexpression and rescue experiments, disproportionately augmenting spontaneous neurotransmission relative to evoked.

### Syb2 Variants Alter SNARE Complex Affinity and Formation.

To delineate the mechanism by which Syb2 variants G73W and E78A drive spontaneous release to levels greatly surpassing wild type, while other variants cannot rescue release, we investigated their interactions with SNARE complex proteins and binding partners.

**Fig. 4.**
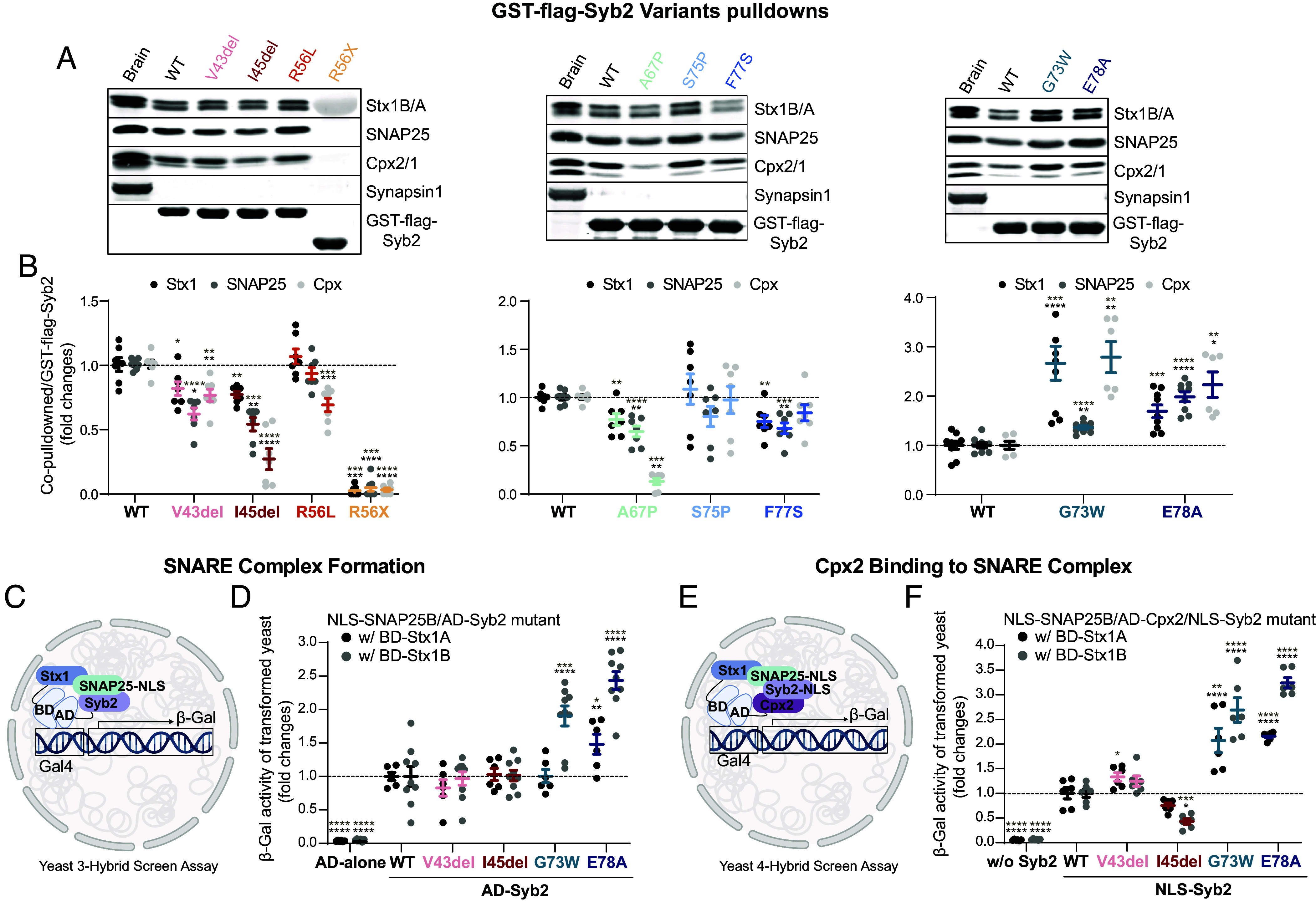
Syb2 patient variants alter SNARE complex affinity and subsequent formation. (*A*) Western blots of GST-flag-Syb2 patient variant co-immunoprecipitation with SNARE complex and auxiliary proteins. (*B*) Recombinant GST-flag-Syb2 cytosolic fraction was added to rat brain homogenate and SNARE complex formation assessed by quantifying co-pull-down with syntaxin1A/B, SNAP25, complexin1/2, and synapsin 1 (negative control) (normalized to GST-flag-Syb2). (*C*) Schematic of yeast 3-hybrid interaction assay to quantify the formation of SNARE complexes composed of patient variant Syb2, SNAP25, and either syntaxin1 A (Stx1A) or syntaxin1 B (Stx1B). HF7c yeast reporter were transformed by plasmids that express GAL4 DNA binding domain-fused syntaxin1 cytosolic fraction (BD-Stx1), GAL4 DNA activating domain-fused Syb2 cytosolic fraction (AD-Syb2), and nuclear localization signal-fused soluble form of SNAP25 (NLS-SNAP25). (*D*) Quantification of β-galactosidase activity measuring SNARE complex formation with both syntaxin1 A and B separately. (*E*) Schematic of yeast 4-hybrid interaction assay to quantify the formation of SNARE complexes composed of patient variant Syb2, SNAP25, complexin2, and either syntaxin1 A (Stx1A) or syntaxin1 B (Stx1B). HF7c yeast reporter were transformed by plasmids that express GAL4 DNA binding domain-fused syntaxin1 cytosolic fraction (BD-Stx1), GAL4 DNA activating domain-fused Complexin2 (AD-Cpx2), nuclear localization signal-fused soluble form of SNAP25 (NLS-SNAP25) and nuclear localization signal-fused Syb2 cytosolic fraction (NLS-Syb2). (*F*) Quantification of β-galactosidase activity measuring SNARE complex formation with both syntaxin1 A and B separately. Values are mean ± SEM. Significance reported as *P < 0.05, **P < 0.01, ***P < 0.001, and ****P < 0.0001 in black for multiple comparison analysis and in brown for pairwise comparisons between each variant and WT. Exact p-values, n numbers, and additional statistical information are provided in Dataset S2.

To quantify Syb2 variant affinity with SNARE complex proteins (syntaxin1 A/B, SNAP25) and complexin1/2, we conducted GST-pull-downs from brain lysate (Fig. 4*A*). Complexin has an unequivocal role in regulating release with both promoting and inhibiting functions (31, 32). V43del, I45del, R56X, A67P, and F77S all had decreased binding to syntaxin1, SNAP25, and complexin1/2 (except F77S for complexin), and consequently, were not able to rescue evoked or spontaneous release in Syb2^−/−^ neurons (Fig. 4*B*). Interestingly, both R56L and S75P had milder phenotypes in Syb2^−/−^ spontaneous neurotransmission rescue experiments and showed minor alterations in protein affinity, suggesting other elements of SNARE complex structure or interactions could be disrupted. Conversely, both G73W and E78A increased binding to syntaxin1, SNAP25, and complexin1/2 (Fig. 4*B*), providing strong correspondence between synaptic vesicle exocytosis and the ability of Syb2 to bind its release partners. Taken together these results highlight how the intricate inner workings of SNARE complex structure are uniquely defined by individual residues within Syb2, in that mutations one residue apart create opposite phenotypes.

To further elucidate the biochemical substrates for release abnormalities, we focused on two N-terminal mutations (V43del and I45del) that cannot rescue release and C-terminal variants (G73W and E78A) that augment spontaneous neurotransmission. While GST-Syb2 pull-downs in brain lysate quantified protein affinity, how the SNARE complex assembles as a unit is fundamental in executing release. To probe Syb2 efficacy in SNARE complex formation in an isolated system, we employed a yeast 3 and 4 hybrid screen assay (with and without complexin) (Fig. 4 *C* and *E*), whereby the amount of β-galactosidase activity is dependent on SNARE complex formation. A caveat to this experimental readout is it does not consider the temporal element of SNARE complex formation and subsequent release, potentially fostering a mismatch between functional readouts of release and β-galactosidase activity. However, G73W and E78A have increased SNARE complex formation, both with and without complexin (Fig. 4 *D* and *F*), highlighting the strength and affinity for SNARE complex formation in the variant genetic background.

These data illustrate how lowered affinities between SNARE complex proteins and complexin stunt exocytosis. However, why an increased affinity and efficacy in SNARE complex formation, specifically in the E78A variant, exceedingly increases spontaneous release, while evoked release is only mildly affected, remains unclear. The observation that a ubiquitous mutation drives abnormal release predominately in one mode of neurotransmission is hard to reconcile with the premise that Syb2 is critical for both evoked and spontaneous release (5). Therefore, we further investigated the structure–function relationship specifically in Syb2 variants G73W and E78A.

### Preservation of Release in Select Variants, Despite Compromised SNARE Complex Structural Integrity.

**Fig. 5.**
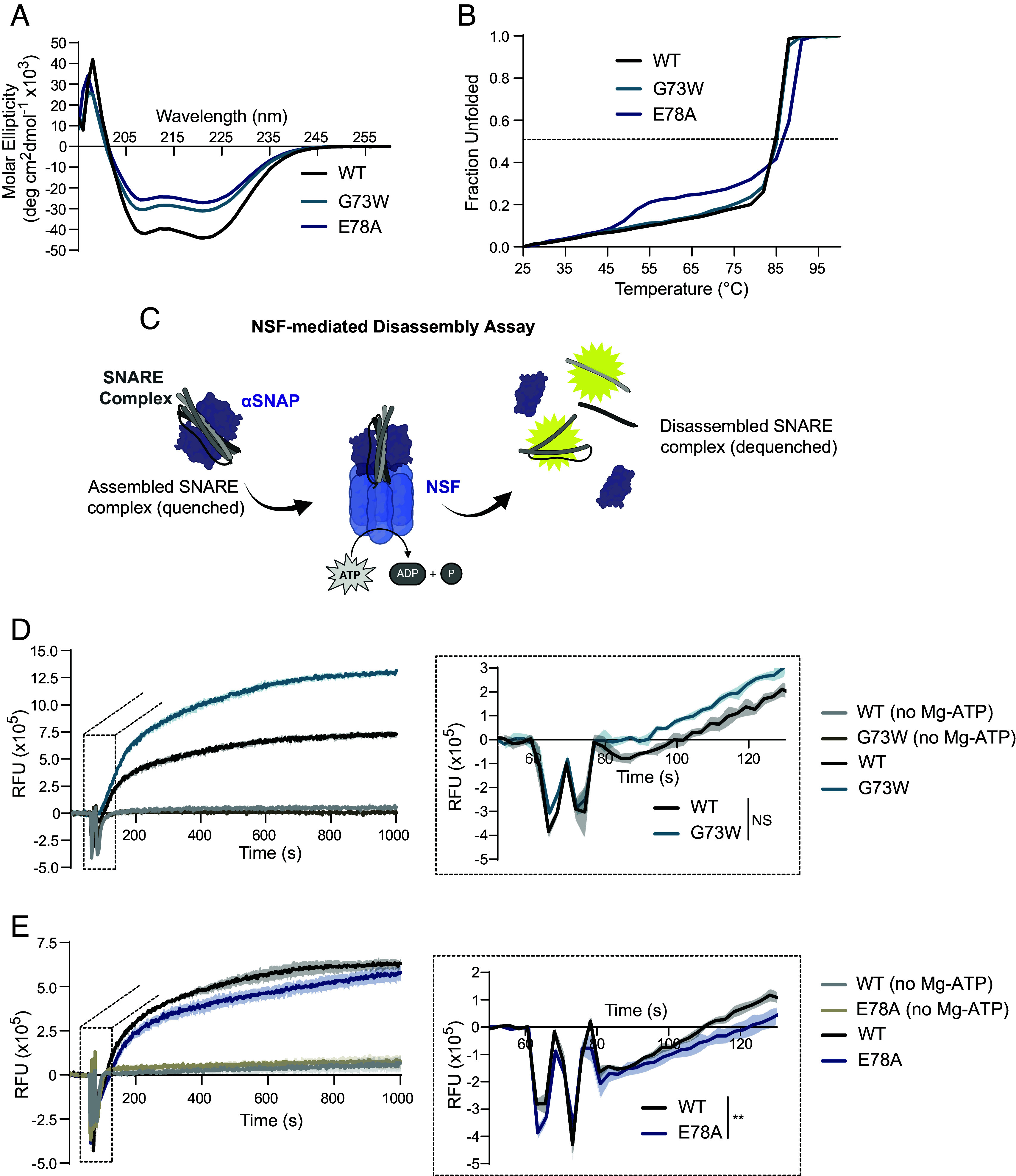
Preservation of release in select variants despite compromised SNARE complex structural integrity. (*A*) Circular dichroism spectra of SNARE complexes with SNAP25A_7–83_,_141–204_, Syb2_28–89_, Syntaxin1A_191–256_ of WT, G73W, and E78A Syb2. (*B*) Thermal melting curves of SNARE complexes observed at 220 nm. (*C*) Schematic of the NSF-mediated disassembly assay measuring bulk fluorescence dequenching upon disassembly of the ternary SNARE complex. The disassembly reaction was initiated by the addition of magnesium. (*D*) NSF-mediated disassembly kinetics of the SNARE complex with WT Syb2 and G73W Syb2, with and without Mg-ATP to initiate NSF mediated activity and an enlargement of the region used to calculate the initial rate of disassembly (90 to 130 s). Of note, the labeling efficiency was higher in the G73W variant. (*E*) NSF-mediated disassembly kinetics of the SNARE complex with WT Syb2 and E78A Syb2, with and without Mg-ATP to initiate NSF mediated activity and an enlargement of the region used to calculate the initial rate of disassembly (90 to 130 s). Values are mean ± SEM, except panel *B* is only mean. Significance reported as *P < 0.05, **P < 0.01, ***P < 0.001, and ****P < 0.0001 in black for multiple comparison analysis and in brown for pairwise comparisons between each variant and WT. Exact *P*-values, n numbers, and additional statistical information are provided in Dataset S2.

The aforementioned biochemical data provides insight into how each Syb2 variant interacts with other SNARE and auxiliary proteins, however, the integrity of the SNARE complex as a unit is integral to exocytosis. Circular dichroism wavelength scans suggest that the G73W and E78A variants form helical bundles similar to the wildtype complex, albeit with altered helical propensities. Using wildtype constructs for syntaxin1 and SNAP-25, the Syb2 variants decrease helical propensity (Fig. 5*A* and *SI Appendix*, Fig. S5*A*). The helical nature of these complexes changes slightly with marginally different constructs and the introduction of cysteine mutations for labeling studies (see below), which shifts α-helical conformations (Fig. 5*A* and *SI Appendix*, Figs. S5*A* and S6*A*). When protein unfolding is assessed, the G73W variant SNARE complex’s melting curve is identical to that of the wild-type SNARE complex. In contrast, the E78A variant SNARE complex has a biphasic behavior, with a slightly higher temperature required for complete denaturation, revealing a more complex melting curve (Fig. 5*B* and *SI Appendix*, Figs. S5*B* and S6*B*). G73W does not change, and E78A slightly increases the temperature at which the SNARE complex loses α-helicity and unfolds, suggesting the complex is stable despite the presence of Syb2 variants. These data highlight the unique structure function relationship of these two anomalous variants, where despite altered coiled-coil structure, the SNARE complex is still stable, and ultimately sufficient for exocytosis.

How this tight SNARE complex effects the equilibrium of assembled and disassembled SNARE complexes is dependent on interactions with the NSF-mediated disassembly machinery (NSF and αSNAP), as well as Munc13 (33, 34). To assess this, the bulk fluorescence of SNARE proteins labeled with fluorophores was quantified as the SNARE complex disassembles from the quenched (assembled) to the de-quenched (disassembled) state (Fig. 5*C*) (35). The initial slopes (90 to 130 s) of this disassembly activity of WT relative to G73W and E78A revealed that the Syb2 E78A variant had a slightly slower initial disassembly activity, whereas the rate of G73W was comparable to the WT complex (Fig. 5 *D* and *E*, replicated experiments in *SI Appendix*, Fig. S6 *C* and *D*). This is consistent with CD scans conducted using the disassembly assay SNARE protein constructs as the Syb2 E78A variant has the highest helicity (*SI Appendix*, Fig. S6*A*). The slightly decreased rate of SNARE complex disassembly could be due to E78A’s increased thermal stability. It is also possible that this mutation makes the SNARE complex not as easily recognizable by αSNAP, decreasing the probability of disassembly. More importantly, the G73W and E78A Syb2 variants appear to form viable SNARE complexes, suggesting that other factors cause these mutations to drive abnormal release predominately in one mode of neurotransmission. For example, the increased complexin binding of the mutants, especially E78A (Fig. 4*F*), may interfere with SNARE complex disassembly (36).

### LLPS-Mediated RIM Exclusion Zone Shapes Syb2 Patient Variant Synaptic Phenotype.

**Fig. 6.**
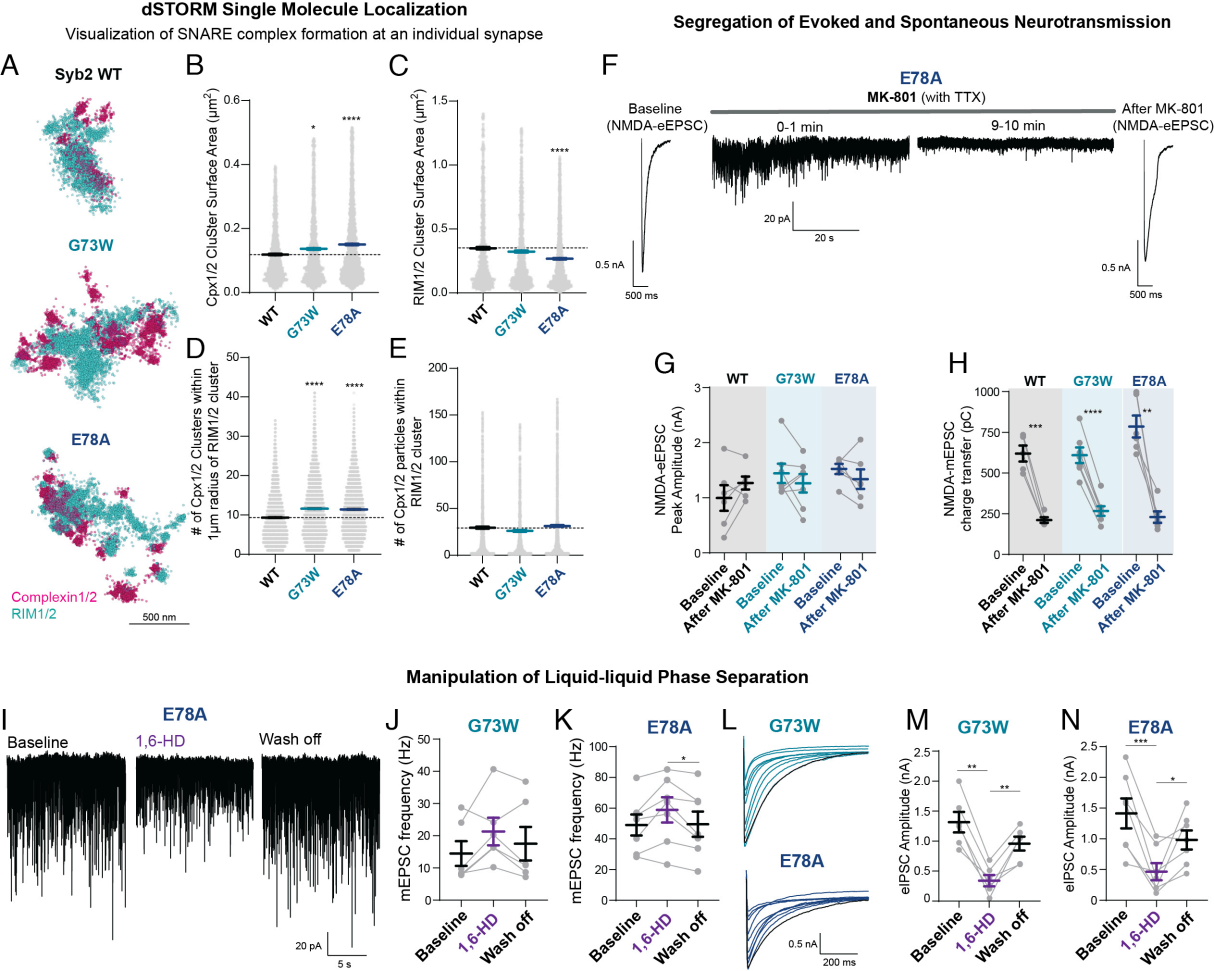
LLPS-mediated RIM exclusion zones shape Syb2 patient variant synaptic phenotype. (*A*) dSTORM single molecules localizations of identified pre-synaptic clusters using DBSCAN and proximity based analysis following WT Syb2 or patient variant Syb2 overexpression on a WT background. (*B*) Of defined synapses complexin cluster and (*C*) RIM1/2 cluster surface area were quantified. (*D*) The number of complexin clusters within a 1 µm radius of a RIM cluster. (*E*) Individual complexin single molecules localized within the α-hull of a RIM cluster. (*F*) Example traces of variant E78A overexpressed in a WT genetic background probing the segregation of evoked and spontaneous neurotransmission by first measuring an evoked NMDA-eEPSC at baseline, washing on TTX and MK-801 to block all NMDARs open during a 10-min spontaneous neurotransmission treatment, rapidly washing off TTX and MK-801, then subsequently measuring an evoked NMDA-eEPSC post treatment. (*G*) Quantification of NMDA-eEPSC amplitude (average of 10 stim at 0.1 Hz) and (*H*) NMDA mEPSC charge transfer (15 s in length) at baseline and after MK-801 treatment in WT, G73W and E78A genetic backgrounds. (*I*) Representative traces of variant E78A overexpressed in a WT genetic background during aliphatic alcohol treatment. (*J*) Quantification of 2 min 3% 1,6-HD treatment on mEPSC frequency in Syb2 G73W and (*K*) E78A patient variants. (*L*) Representative traces of G73W and E78A Syb2 variant eIPSCs during 1,6-HD application at 0.1 Hz with baseline trace in black. (*M*) Quantification of eIPSC amplitude at baseline, following 1,6-HD application, and wash off for G73W and (*N*) E78A (all amplitudes normalized to 1st stim of baseline). Values are mean ± SEM. Significance reported as *P < 0.05, **P < 0.01, ***P < 0.001, and ****P < 0.0001. Exact *P*-values, n numbers, and additional statistical information are provided in Dataset S2.

Evoked and spontaneous release processes are spatially segregated within the active zone (25, 37–45). This segregation is in part mediated by the preferential maintenance of evoked release within pre-synaptic RIM1/2 nanoclusters, where presynaptic sites of fusion are aligned with postsynaptic receptor clusters via synaptic cell adhesion complexes, creating trans-synaptic nanocolumns (46, 47). With the Syb2 variants of interest, we visualized where release is happening within an individual synapse to elucidate if the divergent functional phenotype of evoked vs. spontaneous release is a consequence of synaptic structural constraints. Using super-resolution microscopy [direct STochastic Optical Reconstruction Microscopy (dSTORM)], we visualized active zone organization with RIM1/2 staining and SNARE complexes with complexin staining. Since complexin is a late SNARE priming protein (9, 10), necessary for release, and expressed at relatively low levels within the synapse (molecules/synapse: complexin1/2 ~2,500; Syb2 ~26,000) (48), it served as an excellent marker of SNARE complexes to visualize sites of fusion (Fig. 6*A*).

Overall, individual complexin cluster surface area (composed of numerous single molecule localizations) is increased in G73W and E78A variants, consistent with the increased complexin binding elicited by these variants (Figs. 4 *B* and *F* and 6*B*). On average, the surface area of a complexin cluster was 0.1 µm^2^ and a RIM1/2 cluster 0.35 µm^2^. The size of complexin clusters moderately correlates with RIM1/2 scaffolding size, as I45del had an increased RIM surface area, whereas E78A RIM cluster surface area is decreased (Fig. 6*C* and *SI Appendix*, Fig. S7*C*).

When we probed the spatial organization of complexin relative to RIM clusters, the number of individual complexin clusters within a 1 µm radius of a RIM cluster (denoting the same synapse) significantly increased from 9 in WT to 11 in G73W and E78A, producing on average two more putative release sites per synapse, likely corresponding to sites of spontaneous release (Fig. 6*D*). While the number of complexin single molecule localizations within the RIM1/2 volume was unchanged (Fig. 6*E*). This result suggests that the increased complexin cluster area and total number of clusters in G73W and E78A are largely outside of RIM active zone scaffolding, accounting for the disproportionate augmentation of spontaneous neurotransmitter release. In contrast, two variants that did not augment spontaneous release (V43del and I45del) did not cause a similar biased localization of complexin puncta outside of RIM clusters, attesting the specificity of this observation to variants that disproportionately augment spontaneous release (*SI Appendix*, Fig. S7 *A*–*E*).

Despite the increased affinity for SNARE complex formation in variants G73W and E78A, the active zone marked by RIM clusters is limiting the number of exocytotic complexes that can form within it, thereby potentially setting a threshold for evoked release by creating a distinct exclusive “evoked release zone.” This is consistent with a lack of augmented evoked release in other characterized variants of core SNARE complex components (49). Our model proposes that despite the increase of SNARE complexes within a single synapse in G73W and E78A, excess complexes are barred from the RIM exclusion zone, disproportionately upregulating minis.

To further evaluate whether this high spontaneous release frequency (~64 Hz) encroaches on the location of evoked release, we functionally probed the spatial segregation of evoked and spontaneous release. For this purpose, we utilized the NMDAR use dependent pore blocker, MK-801, which only blocks NMDARs in the open state (37). We initially measured the amplitude of NMDA-eEPSCs, subsequently washed on MK-801 for 10 min in the presence of TTX to block spontaneous neurotransmission, then measured the remaining NMDA-eEPSC response (Fig. 6*F*). Remarkably, despite the robust blockade of NMDA-mEPSCs, the subsequent evoked response detected in neurons expressing WT, G73W, and E78A variant Syb2 were unaltered (Fig. 6 *G* and *H*). This indicates that spontaneous neurotransmission, no matter how high the frequency, does not infringe on the location of evoked release within the synapse, underscoring the existence of clear boundaries.

Next, we probed the nature of this demarcation between these two forms of release by evaluating the role of liquid–liquid phase separation (LLPS). LLPS properties of synaptic proteins suggest a framework by which a synapse can compartmentalize a single micron of space into a temporarily and spatially precise molecular machine (50–64). Specifically, phase separation of RIM1/2 preferentially supports evoked release efficacy and fidelity via nanoclustering of the active zone (62). Here, we used the small molecule, 1,6-hexanediol, to selectivity disrupt RIM1/2 LLPS mediated nanoclustering in the G73W and E78A genetic backgrounds (Fig. 6*I*). Upon acute disruption of spatial organization, augmented spontaneous release persisted (Fig. 6 *I*–*K*), however, when evoked release is probed, there is a rapid decrease in eIPSC amplitude (Fig. 6 *L*–*N*), suggesting that the phase separation of RIM nanoclusters is not a necessary structural component of spontaneous release. This is consistent with the model that exorbitant spontaneous release occurs outside RIM clusters, suggesting that RIM LLPS creates an exclusion zone within the synapse, where SNARE complex formation is spatially confined and regulated, regardless of its propensity to assemble. Therefore, the ability of the E78A and G73W genetic variants to drive excessive fusion is limited to spontaneous release and largely spares evoked neurotransmission due to the nano-level structural confines of phase separation-induced boundaries.

## Discussion

The investigation of Syb2 SNAREopathies enables the association between specific residues in Syb2 with molecular alterations, functional synaptic changes, and neurological symptoms, generating a comprehensive approach to study the biological hierarchy of SNAREopathies. Seven of the nine variants assessed display negative synaptic phenotypes that largely downregulate release across all modes of neurotransmission. Conversely, two variants, G73W and E78A, are dominant positive, providing a particularly powerful disease-relevant genetic system to study pre-synaptic function, as spontaneous release is drastically augmented and evoked release is mildly altered. These two dominant positive variants reveal that while SNARE complex integrity is the molecular substrate for disease, the actual synaptic phenotype is shaped by the nano-level structural organization of the synapse, ultimately driving the pathophysiology.

Most proteins involved in exocytosis, with some notable exceptions (65), are shared between different modes of release, with the loss of Syb2 resulting in impairments in both evoked and spontaneous neurotransmission (5). However, the G73W and E78A variants of Syb2 demonstrate a distinct divergence: Spontaneous release is significantly enhanced, while evoked release is only mildly dysregulated. Remarkably, despite the extreme rate of spontaneous release with Syb2 E78A, the fundamental segregation of release modes remains intact, with evoked and spontaneous release operating independently of one another. Although the G73W and E78A variants enhance SNARE complex assembly and the binding to complexin, the location of these complexes is excluded from RIM hotspots for evoked release, resulting in a predominantly unidimensional synaptic phenotype of augmented spontaneous release. Here, our use of complexin staining to visualize the localization of SNARE complexes was a novel approach. We posit that this approach is supported by the following points. First, cells were permeabilized in 0.2% Triton-X for 30 min. We believe this treatment is sufficiently robust to wash off a significant portion of the soluble fraction of complexins within the terminals. Second, our analysis was focused on complexin clusters in the vicinity of RIM clusters. We found that the total area covered by RIM or complexin staining were comparable, although their relative distributions were altered. This is important because RIM has a very low copy number per synapse (~50), whereas total complexin copy number is around 2,500 (48). This finding supports the above premise that a large fraction of soluble complexin were removed during the permeabilization process. Finally, under the same experimental conditions, the relative distribution of RIM versus complexin clusters showed significant differences between variants with enhanced spontaneous release (G73W, and E78A) and variants without (V43del, I45del) (Fig. 6 and *SI Appendix*, Fig. S7). These results further attest to the specificity of complexin localization with respect to RIM nano-clusters marking sites of SNARE complexes.

Since G73W and E78A both have an increased release probability in the Syb2^−/−^ background, these variants can function within the RIM nanocolumn, but their impact on nanocolumn-restricted evoked release is overshadowed by augmented extra-columnar spontaneous release. We cannot exclude a direct impact of these variants on the fusion energy landscape, as two previously studied SNAP25 variants that augment spontaneous release and increase release probability both lower the energy barrier for fusion (66). This electrophysiology phenotype is similar to both Syb2 variants G73W and E78A, suggesting variant-driven changes in the fusion energy landscape could contribute to the observed synaptic phenotypes. Together, this suggests various alterations along the neurotransmitter release pathway contribute to the multifaceted nature of SNAREopathies. Nevertheless, the observed predominant effect on spontaneous release suggests a consistent gain of function phenotype. LLPS appears to mediate this boundary by creating hydrophobic phase separated condensates, which restrict the extent of evoked release, potentially by regulating the quantity of proteins permitted in the evoked exclusion zone (62). These results are also consistent with the recent cryo-ET imaging results that show membrane-proximal synaptic vesicles are offset, or adjacent to, trans-synaptic nanocolumns, but not on top of the nanocolumns (67), suggesting the pre-synaptic scaffolding complex is a restricted area with a limited synaptic vesicle population. Thus, spatial organization may serve as a mechanism to dynamically align and control synaptic vesicles within the active zone, thereby organizing hotspots for evoked neurotransmitter release. In this way, spatial organization could play a crucial role in regulating synaptic transmission by influencing release parameters, such as release probability, independent of the SNARE complex’s propensity to assemble. However, a fundamental question remains of the mechanisms that limit the size of these LLPS condensates (62). The patient SNARE variants bolster the framework that neurotransmission is more than an all-or-nothing process but operates within the nano-architecture of the synapse, with numerous molecular, structural, and chemical regulatory elements that order the precise timing and location of neurotransmitter release.

In this study, we used Syb2 variants to uncover the convergence of synaptic disease mechanisms across SNAREopathies. Of the first five Syb2 SNAREopathy patients identified, C-terminal domain patients variants (S75P, F77S, and E78A) had more severe neurological impairments than the N-terminal domain deletion mutants (V43del, I45del) (Table 1) (19, 49). This clinical classification is consistent with the electrophysiological results observed here, with C-terminal variants having a more robust neurotransmission deficit compared to N-terminal variants. However, with a pleiotropic disease, the broad classification of disease severity is not as effective for treatment development, as F77S and E78A resulted in similar severities of disease but have different underlying mechanisms. Therefore, accumulating evidence from multiple studies indicates that SNAREopathies should be classified not by protein identity, but by their specific neurotransmission deficits, which can then be directly targeted for treatment. Recently, potassium channel inhibitors [4‐aminopyridine (4‐AP) and 3,4‐diaminopyridine (DAP)] were used to delay repolarization following an action potential, increasing release in select Syt-1 and Syb2 SNAREopathies, and excitingly, improving symptoms in a patient (Syb2 R56X) (18, 20). As the number of identified patients with SNAREopathies increases, elucidating the synaptic underpinnings becomes crucial.

Our results strengthen the conclusion that aberrant spontaneous neurotransmission is sufficient to drive disease exemplified by Syb2 variants G73W and E78A. Across the Syb2 and SNAP25 SNAREopathies studied, five variants severely dysregulate spontaneous release relative to evoked (Syb2 variants G73W and E78A, SNAP25 variants L50S, V48F and D166Y) (3). While the molecular underpinnings of augmented spontaneous release are different, the synaptic outcome is similar. Thus, augmented spontaneous neurotransmission is a shared functional sub-class within SNAREopathies and provides a potential target for therapeutic intervention. The role of aberrant spontaneous neurotransmission in neurological disease as well as ketamine’s rapid antidepressant action arising from spontaneous release modulation reflect the utility of spontaneous neurotransmission at the synapse (3, 68, 69). Whereby in two separate clinical populations, one with neurodevelopmental encephalopathies and one with major depression disorder, spontaneous neurotransmission is a key molecular substrate. Ultimately revealing spontaneous neurotransmission as an autonomous mode of release involved in a broad range of functions.

Syb2 SNAREopathies highlight the nanoscale layers of regulation that orchestrate synaptic signaling, where regardless of SNARE complex propensity to assemble, release is still specifically localized and compartmentalized. The dynamics governing precise neurotransmission are driven by the balance of SNARE proteins operating within the confines of presynaptic structure. Taken together, presynaptic nanoclusters (and potentially, trans-synaptic nanocolumns) utilize active zone phase separation as a dynamic regulator of synaptic organization, concurrently conducting multiple modes of release within a single micron. The investigation into Syb2 variants not only provides mechanistic insight into rare diseases but unveils basic synaptic physiology that governs the nano-environment of the synapse.

## Materials and Methods

Primary hippocampal and embryonic mouse brain cultures were prepared, with genotype confirmation by tail genotyping, and HEK293 cells were used for lentivirus production. Experimental procedures included immunocytochemistry, dSTORM super-resolution microscopy, electrophysiological recordings using whole-cell patch-clamp techniques, and viral transfection. Protein studies involved SDS-PAGE, Western blotting, GST pull-downs, yeast interaction assays, and purification of SNARE proteins, NSF, and αSNAP, followed by functional assays including fluorescence dequenching for SNARE complex disassembly and circular dichroism for secondary structure analysis. Drug treatments with reagents such as 1,6-hexanediol and MK-801 were used for studying LLPS and segregation of NMDARs responding to evoked versus spontaneous release. Statistical analysis encompassed outlier identification, assessment of data normality, and utilization of appropriate parametric or nonparametric tests, with significant differences determined at *P* < 0.05 and data reported as mean ± SEM, as described in more detail in associated datasets and figures. For more detailed experimental procedures, please refer to *SI Appendix*.

## Supplementary Material

Appendix 01 (PDF)

Dataset S01 (XLSX)

Dataset S02 (XLSX)

Dataset S03 (XLSX)

## Data Availability

Plasmids will be available upon request from the lead contact or from Addgene (https://www.addgene.org/Ege_Kavalali/) (70). Synaptobrevin2 knock-out mice are available from the Jackson Laboratory (https://www.jax.org/strain/006380) (71). All data are included in source data excel documents for main figures (Dataset S1), for statistical analysis (Dataset S2) and supplementary figures (Dataset S3) are included within the paper. This paper does not contain any original code. All other data are included in the manuscript and/or supporting information.
