# Supplementary material for: Synaptobrevin-2 disease variants reveal spatial constraints within the presynaptic active zone
Source: Proc Natl Acad Sci U S A. 2025 Oct 30;122(44):e2507347122. doi: 10.1073/pnas.2507347122 (PMC12595502; doi:10.1073/pnas.2507347122)
Supplement: Supplementary file 1 — Appendix 01 (PDF) [file pnas.2507347122.sapp.pdf]

PNAS

Supporting Information for
Synaptobrevin-2 Disease Variants Reveal Spatial Constraints Within the Presynaptic Active Zone

Natalie J. Guzikowski[1,2], Elena D. Bagatelas[1,2], Ok-Ho Shin[1,2], Yousuf A. Khan[3-7], Luis Esquivies[3-7], Baris Alten[1,2], Axel T. Brunger[3-7] and Ege T. Kavalali*[1,2]

Ege T. Kavalali
Email: ege.kavalali@vanderbilt.edu

This PDF file includes:

Supplemental Materials and Methods

Figures S1 to S7

MATERIALS AND METHODS

Primary culture

Primary hippocampal cultures for Syb2 variant overexpression on WT background were prepared from P1/2 wildtype rat pups of either sex per standard protocols. Embryonic cultures for Syb2 variant rescue on KO background were prepared from E18-20 mouse pups of either sex per standard protocols (KO, Het, and WT littermate mouse embryos were cultured) (1). For breeding, a male Syb2$^{+/-}$ and female Syb2$^{+/-}$ mouse were paired and housed together. Embryos were surgically obtained from the pregnant dams to generate neuronal cultures.

To generate primary cultures, pups were rapidly decapitated at the head neck junction and the hippocampus (WT P1/2 pups) or the hippocampus/cortex (E18-20 pups) were isolated from each hemisphere in cold 20% FBS containing Hank's balanced salt solution. The hippocampus and cortex were both collected from embryonic pups to increase brain tissue yield per pup due to the low probability of KO animals. The tail from each embryonic pup was collected and genotyped to confirm genotype.

All steps after WT hippocampi isolation or embryonic cortex/hippocampi isolation are the same for culturing and plating. Briefly, the tissue was treated with 10 mg/mL of trypsin and 0.5 mg/mL DNase solution for 10 minutes at 37°C. Following trypsinization, tissue was mechanically dissociated in 0.5 mg/mL DNase, then spun down at down at 250 RCF for ten minutes to isolate a cell pellet. Neurons were resuspended in plating media and directly added to 50:1 MEM:Matrigel coated coverslips. Neurons were plated at a ratio of 1 pup to 10 coverslips in media containing MEM (no phenol red), 5 g/D-glucose, 0.2 g/L NaHCO3, 0.1 g/L transferrin, 10% FBS, 2 mM L-glutamine and 20 mg/L insulin. No. 0 glass coverslips were used for electrophysiology and No. 1.5H MatTek glass bottom dishes for dSTORM. 24 hours post plating, media was changed to growth media containing 5% FBS, 0.5 mM L-glutamine, no insulin, and the addition of B27 supplement and 4 mM cytosine arabinoside. On DIV 4, media was changed to a reduced concentration cytosine arabinoside media at 2 mM. Neurons were maintained in incubators at 5% CO_2 37°C until experiments were conducted between DIV 14-18.

All experiments were conducted in accordance with Institutional Animal Care and Use Committee (Protocol ID: M1800103-02) guidelines and procedures at Vanderbilt University.

HEK 293 Cell Lines

Human embryonic kidney-293 (HEK293) cells were used to generate lentivirus for neuronal infection. HEK293 cells were maintained in Dulbecco's Modified Eagle Medium supplemented with 10% FBS and penicillin and streptavidin out of 500 mL in incubators at 5% CO_2 37°C. HEK293 cells were passaged and split at ~75% confluency.

Immunocytochemistry

Immunohistochemistry was performed between DIV 14-18 in WT cultures overexpressing WT, V43del, I45del, G73W, and E78A Syb2 constructs. To examine basal properties of synaptic organization in each group, samples were rapidly fixed with 4% PFA 4% sucrose solution for 20 minutes at RT. Neurons were then permeabilized with 0.2% Triton-X for 30 minutes at RT. Following permeabilization, neurons were blocked with a 2% BSA, 1%

goat serum, 1% donkey serum blocking solution. Samples were then incubated overnight at 4°C in primary antibody diluted in blocking buffer at the following concentrations: anti-RIM1/2 (GP) (1:500 Synaptic Systems 140 205, lot: 1-5) and anti-Complexin1/2 (R) (1:750 Synaptic Systems 122 002, lot:1-16). The next day, samples were then incubated in secondary antibody (AF647 anti-rabbit: Invitrogen A31573, lot: 2359136; CF568 anti-guinea pig Biotium 20492) for 90 minutes at RT. Samples were then treated with a 4% PFA 4% sucrose solution for 20 minutes at room temperature as a post-staining fixation step. Samples were stored in storage buffer and kept at 4°C in the dark until imaging.

dSTORM Microscopy

dSTORM imaging, single molecule localization and analysis were conducted on the Vutara VXL Comprehensive Workstation (microscope and software). Imaging was conducted with a 555 nm and a 640 nm excitation laser with biplane illumination, a 60x objective (1.3 NA, silicone oil), and a sCMOS (scientific Complementary metal–oxide–semiconductor) detector. Samples were imaged for 2 hrs at RT in dSTORM imaging buffer containing Cysteamine (MEA), Glucose Oxidase, and Catalase (2, 3).

20,000 images were acquired per channel at 50 frames per seconds (20 ms exposure time) at a single z-plane of ~2 µm thickness. For fluorophore localization, 3D measured point spread functions collected during microscope calibration with B-Spline PSF Interpolation method for PSF fitting of localizations was used.

Following localization, individual single molecule localizations (SMLs) were filtered to select only SMLs with a radial precision ≤ 25 nm and axial precision ≤ 55 nm. To define clusters, DBScan, Density-Based Spatial Clustering of Application with Noise, was used to cluster each probe independently with the following parameters: maximum particle distance of 0.1 µm, minimum particle count of 30, and surface hull α shape radius of 0.15 µm. The use of DBScan allowed us to define arbitrarily shaped clusters, grouping SMLs into a cluster based on density not shape.

Following the classification of RIM1/2 and Complexin1/2 clusters, colocalization was performed to only include clusters with both probes present to identify a synapse. From this point, individual cluster surface areas were calculated of probes with hull overlap and the number of complexin1/2 SMLs located within the RIM1/2 α-hull. Separately, the number of complexin clusters located within 1 µm of a RIM cluster were quantified (more clusters identified as not all had hull overlap). Discrete clusters were counted as their surface hull α shapes defined clear cluster boundaries, resulting in numerous separable clusters localized at an individual synapse. The Vutara VXL Comprehensive Workstation software was used to create example dSTORM images displaying single molecule localizations.

Electrophysiology – whole cell patch clamp

Whole cell voltage clamp electrophysiology was performed with a CV203BU head stage, Axopatch 200B amplifier, Digidata 1320 digitizer, and 8 Clampex 8.0 software (Molecular Devices). Whole cell patch clamp recordings were filtered at 1 kHz and sampled at 100 µs. 3-6 MΩ borosilicate glass patch pipettes were used for all recordings and cells were held at $^{-}$70 mV for all voltage clamp experiments.

External bath solution contained 150 mM NaCl, 4 mM KCl, 10 mM D-glucose, 10 mM HEPES, 1.25 mM $MgCl_2$, and 2 mM $CaCl_2$ at pH 7.4 and 320 mOsM with TTX (1 µM), APV (50 µM), and CNQX (10 µM) to isolate mIPSCs; TTX (1 µM), APV (50 µM), and bicuculline (20 µM) to isolate mEPSCs; APV (50 µM) and CNQX (10 µM) to isolate eIPSCs; APV (50 µM) and bicuculline (20 µM) to isolate eEPSCs. To isolate NMDA currents, magnesium was not included in the external bath solution, and 15 µM glycine and 1 µM strychnine hydrochloride were added. To isolate NMDA-eEPSCs, bicuculline (20 µM) and CNQX (10 µM) were added to the bath. To isolate NMDA-mEPSCs, bicuculline (20 µM), CNQX (10 µM) and TTX (1 µM) were added to the bath. A parallel bipolar electrode provided field stimulation at 35 mA for evoked recordings. For 1,6-HD evoked experiments cells were stimulated at 0.1 Hz for ten stimulations at baseline, 1,6-HD treatment, and wash off respectively. The 10th stim (normalized to 1st stim of baseline) for each treatment group was compared.

For voltage clamp experiments, internal pipette solution contained 115 mM Cs-$MeSO_3$, 10 mM CsCl, 5 mM NaCl, 10 mM HEPES, 0.6 mM EGTA, 20 mM tetraethylammonium-Cl, 4 mM Mg-ATP, 0.3 mM Na_3GTP, and 10 mM QX-314 [N-(2,6-dimethylphenylcarbamoylmethyl)-triethylammonium bromide] at pH 7.35 and 300 mOsm.

Lentiviral infection/transfection

To make lentivirus, HEK 293 cells were transfected with three packaging plasmids pCMV-VSV-G, pMDLg/pRRE, and pRSV-Rev at 0.5 µg each (4, 5), and the plasmid of interest at 1 µg ug for all Syb2 variants and empty vector control (pFUW) with FuGENE 6. HEK media was changed to neuronal growth media 24 hours post-transfection and lentivirus collected 48 hours later. Lentivirus was spun down at 690 RCF for 15 minutes, and then 60 µl of the lentivirus of interest added to neuronal media on DIV4.

SDS-polyacrylamide gel electrophoresis and Western Blotting

Protein for quantification of Syb2 variant overexpression was collected from primary hippocampal cultures infected with the respective Syb2 construct in lysis buffer (2x Laemmli sample buffer, 2-Mercaptoethanol, and protease and phosphatase inhibitors (phosSTOP and cOmplete tablets, mini (Roche))).

Samples were sonicated for 40 minutes then heated at 40°C for ten minutes. Samples were loaded into a 10% SDS-PAGE gel and run for ~2hrs at 110 volts. Protein was then transferred with a Trans-Blot Turbo Transfer System to a nitrocellulose membrane. Samples were blocked in 5% milk in Tris-Buffered saline (TBS) for 1hr. Membranes were then incubated overnight in anti-Syb2 (Synaptic systems 104 211 Cl69.1) and anti-SNAP25 (loading control) (Synaptic systems 111 111 Cl71.2) diluted in 5% milk at 4°C overnight.

The following day, membranes were then washed 3 x in TBS for 10 minutes. Membranes were then incubated in secondary antibody goat anti-mouse (LiCOR 926-32210) diluted in 5% milk for 1hr. Following secondary antibody incubation, membranes were washed 3 x in TBS for 10 min. Membranes were imaged with the Odyssey Clix imaging system (Li-Cor). Band intensities were calculated using Fiji image J and normalized to their respective loading control (SNAP25) and then empty vector control. Five sets of samples were run from five independent cultures. Blots were scanned twice due to band intensity differences,

as Syb2 is overexpressed. The truncation mutation, R56X, is not recognized by the Syb2 antibody (per manufacturer: Botox B Syb2 cleavage product (AA 1 – 76) not recognized due to conformational masking).

GST-flag-Syb2pulldowns

Recombinant GST-flag-Syb2 cytosolic fraction was added to rat brain homogenate, final 1% Triton X-100 was added to solubilize membranes and trigger SNARE complex formation composed of GST-flag-Syb2. After 1hr rocking at 4°C, GST-Sepharose beads were added to pulldown GST-flag-Syb2 complex, washed 5 times with buffer, and co-pulldowned proteins were quantified with SDS-polyacrylamide gel electrophoresis (SDS-PAGE) and Western blotting. Western blot protocol the same as detailed above.

Co-pulldowned proteins were normalized to GST-flag-Syb2. Antibodies used were: Syntaxin 1B and A = Stx1B/A (Synaptic Systems 110011 Cl78.2), SNAP25 (Synaptic Systems 111 111 Cl71.2), Complexin 2 and 1 = Cpx2/1 (Synaptic Systems 122 002), Synapsin1 (Synaptic Systems 106 011 Cl46.1)), and Flag monoclonal antibody (Invitrogen MA1-91878).

Yeast interaction assay

Yeast 3-hybrid interaction assay: HF7c yeast reporter was transformed by plasmids that express GAL4 DNA binding domain-fused syntaxin 1 cytosolic fraction (BD-Stx1), GAL4 DNA activating domain-fused Syb2 cytosolic fraction (AD-Syb2), and nuclear localization signal-fused soluble form of SNAP25 (NLS-SNAP25). Soluble SNAP25 was generated by C85S, C88S, C90S, and C92S mutations to prevent the palmitoylation of SNAP25. After 3 days of cultivation on a selection agar plate, 6-9 independently transformed yeast colonies were cultured in a liquid medium and β-galactosidase activity was measured by employing o-Nitrophenyl-beta-D-galactopyranoside (ONPG) as a substrate(6).

Yeast 4-hybrid interaction assay: HF7c yeast reporter was transformed by plasmids that express BD-Stx1, NLS-SNAP25, NLS-Syb2, and AD-Cpx2. Beta-galactosidase activity was measured as described above. GAL4 DNA binding domain-fused syntaxin1 cytosolic fraction (BD-Stx1), GAL4 DNA activating domain-fused Complexin2 (AD-Cpx2), nuclear localization signal-fused soluble form of SNAP25 (NLS-SNAP25) and nuclear localization signal-fused Syb2 cytosolic fraction (NLS-Syb2).

NSF and αSNAP Expression and Purification

A construct containing the NSF sequence from *C. griseus* was transformed into B,,L21(DE3)-RIL *E. coli* cells. 8 L of auto-induced cells were harvested and subjected to Nickel-NTA chromatography, followed by size exclusion chromatography (SEC) on a Superdex 200 16/6- column (GE Healthcare). Fractions containing NSF were monomerized by incubating with sodium phosphate buffer overnight. This monomeric NSF was then purified with SEC and then reassembled into oligomers by incubation with 1 mM ATP and purified in a final SEC (7). αSNAP from *R. norvegicus* was purified with Nickel-NTA chromatography followed by SEC(35). G73W and E78A Syb2 were generated through the use of a g-block (IDT-DNA).

SNARE Expression, purification, and complex formation

Plasmids were co-transformed into BL21 STAR (DE3) *Escherichia coli* competent cells (Invitrogen), and grown in 4 L of LB autoinducing media at 37° C for 4 hours, followed by 16 hours at 25° C. The cells were spun down at 5,000 RPM and resuspended in 360 mL of lysis buffer (50 mM NaPi 8, 300 mM, 10 mM Imidazole, 0.5 mM TCEP, 1% Triton X-100) supplemented with lysozyme and DNAse I. The cells were lysed by sonication (3 seconds on, 9 seconds off, 60% power) for 5 minutes twice, and the cell lysate was clarified by centrifugation (43,000 RPM for 40 minutes).

The clarified cell lysate was collected and mixed with 5 mL of Nickel beads (Qiagen) previously equilibrated with lysis buffer. The beads were incubated at 4° C for 1-2 hrs, collected and washed with 10 column volumes of wash buffer (50 mM NaPi 8, 30 mM Imidazole, 300 mM NaCl, 0.5 mM TCEP). The complex was eluted by washing the column with elution buffer (50 mM NaPi 8, 400 mM Imidazole, 300 mM NaCl, 0.5 mM TCEP) and collecting 1 mL fractions into Eppendorf tubes containing 2 μL of 0.5 mM EDTA (final EDTA concentration 1 μM). The fractions containing the complex were pooled, Tobacco Etch Virus protease was added to remove the His tag, and the sample was dialyzed overnight at 4° C in 1 L of dialysis buffer (50 mM Tris 8, 250 mM NaCl, 0.5 mM TCEP, 1 mM EDTA) using a 10 kDa MWCO dialysis cassette (Thermo).

After dialysis, the sample was filtered and the NaCl concentration was diluted to 50 mM using Tris pH 8, 0.5 mM TCEP, and 1 mM EDTA. The sample was loaded on to a 4.6/100 MonoQ column (Cytiva) and the complex was purified using a salt gradient (50-500 mM NaCl over 40 CV, Buffer A: 50 mM Tris 8, 50 mM NaCl, 1 mM EDTA, 0.5 mM TCEP; Buffer B: 50 mM Tris 8, 500 mM NaCl, 1 mM EDTA, 0.5 mM TCEP). Fractions containing the complex were concentrated and further purified by loading onto a s200 16/60 column (Cytiva) equilibrated with 50 mM Tris 8, 50 mM NaCl, and 0.5 mM TCEP.

The truncated ternary neuronal complexes used for CD experiments shown in Figure 5 and Supplemental Figure 5 consisted of SNAP-25A (residues 7-83 and 141-204), Syntaxin-1A (residues 191-256), and his-tagged synaptobrevin-2 (residues 28-89). The more complete ternary neuronal complex with cysteine mutations for labeling used for the disassembly assay was also used for CD experiments shown in Supplemental Figure 6A-B and consisted of SNAP-25A (residues 1-209), Syntaxin-1A (residues 1-265, C145S/S249C/K253C), and his-tagged synaptobrevin-2 (residues 1-96). In addition, variant (G73W and E78A Syb2) ternary neuronal complexes were also prepared.

All these complexes were purified with nickel-NTA chromatography followed by MonoQ Ion Exchange Chromatography, and SEC.

<u>Fluorescence Dequenching-based SNARE Disassembly Assay</u>

Reactions were performed essentially as previously described (8, 9). Briefly WT, G73W, and E78A neuronal SNARE complexes were labeled with Oregon Green Maleimide 488 overnight in degassed buffer (50 mM Tris pH 8, 150 mM NaCl, 1 mM TCEP, 1 mM ATP, 1 mM EDTA). 60 μL final volumes were assembled with 83.3 nM NSF, 5.25 μM αSNAP, and 1.75 μM labeled neuronal SNARE complex. Reactions were initiated by trituration in a FlexStation II 384-well plate reader (Molecular Devices). Disassembly rates were calculated using the initial, linear range of disassembly activity.

<u>Circular Dichroism Experiments</u>

Circular dichroism measurements were conducted with circular dichroism spectrometer Model 202–01 (Aviv Biomedical) equipped with a temperature controller. Data were collected with 10 μM samples of WT and mutant complexes in 10 mM Tris-HCl pH 8.0, 100 mM NaCl, 0.5 mM EDTA buffer over a wavelength range of 195–260 nm, with 1 nm increments, in a 1 mm path length cell at 25 °C. Temperature melting scans were performed at a wavelength of 220 nm by increasing the temperature from 25 to 100 °C in 3 °C temperature increments, a 2 min temperature equilibration time, and a 3 s averaging time.

Drug Treatments

For liquid-liquid phase separation experiments, neurons were perfused with the aliphatic alcohol 1,6-hexanediol (Sigma Aldrich 240117) at 3% mass to volume ratio for two minutes. To block NMDAR mediated currents, neurons were perfused with MK-801 at 10 μM for 10 minutes.

Quantification and Statistical Analysis

Each set of experiments were performed at least twice (from two different sets of neuronal cultures, if possible). Mini Analysis software (Synaptosoft) was used to calculate mPSC frequencies, amplitudes, and kinetics. Clampfit 10.3 (Molecular Devices) was used to analyze ePSCs. GraphPad Prism 9 was used to conduct all analysis. First outliers were identified with a robust regression and outlier removal test (ROUT) for all electrophysiology and dSTORM analyses. Then, a Shapiro-Wilk test for normality was run to select the appropriate parametric or nonparametric statistical analysis to compare groups. Multiple comparisons tests have less statistical power, potentially causing significant results to be overshadowed with multiple groups; therefore, both multiple comparisons and pairwise comparison tests were used to analyze the data especially, since the main comparison of scientific interest is Syb2 variant v. control (WT) and each genotype is independent. When comparing two groups a paired or unpaired t-test or nonparametric Mann Whitney test was conducted. When comparing three or more groups a one-way ANOVA or nonparametric Kruskal-Wallis test or Friedman test with Tukey, Dunnett, or Dunn's post hoc multiple comparisons tests was conducted as appropriate. $p < 0.05$ was considered statistically significant. Data presented as mean ± standard error of mean (SEM). For more information, please refer to Dataset S2 detailing statistical test run, n numbers, mean, SEM, and p-values.

REFERENCES

1. S. Schoch, et al., SNARE Function Analyzed in Synaptobrevin/VAMP Knockout Mice. Science 294, 1117–1122 (2001).

2. N. J. Guzikowski, E. T. Kavalali, Super-resolution imaging of synaptic scaffold proteins in rat hippocampal neurons. STAR Protoc. 4, 102080 (2023).

3. L. Martin, et al., A protocol to quantify chromatin compaction with confocal and super-resolution microscopy in cultured cells. STAR Protoc. 2, 100865 (2021).

4. S. A. Stewart, et al., Lentivirus-delivered stable gene silencing by RNAi in primary cells. RNA N. Y. N 9, 493–501 (2003).

5. T. Dull, et al., A third-generation lentivirus vector with a conditional packaging system. J. Virol. 72, 8463–8471 (1998).

6. A. M. Shih, O.-H. Shin, Interactions among the SNARE proteins and complexin analyzed by a yeast four-hybrid assay. Anal. Biochem. 416, 107–111 (2011).

7. M. Zhao, et al., Mechanistic insights into the recycling machine of the SNARE complex. Nature 518, 61–67 (2015).

8. D. J. Cipriano, et al., Processive ATP-driven Substrate Disassembly by the N-Ethylmaleimide-sensitive Factor (NSF) Molecular Machine. J. Biol. Chem. 288, 23436–23445 (2013).

9. Y. Gao, et al., Sensory deficit screen identifies nsf mutation that differentially affects SNARE recycling and quality control. Cell Rep. 42, 112345 (2023).

Supplemental Figures

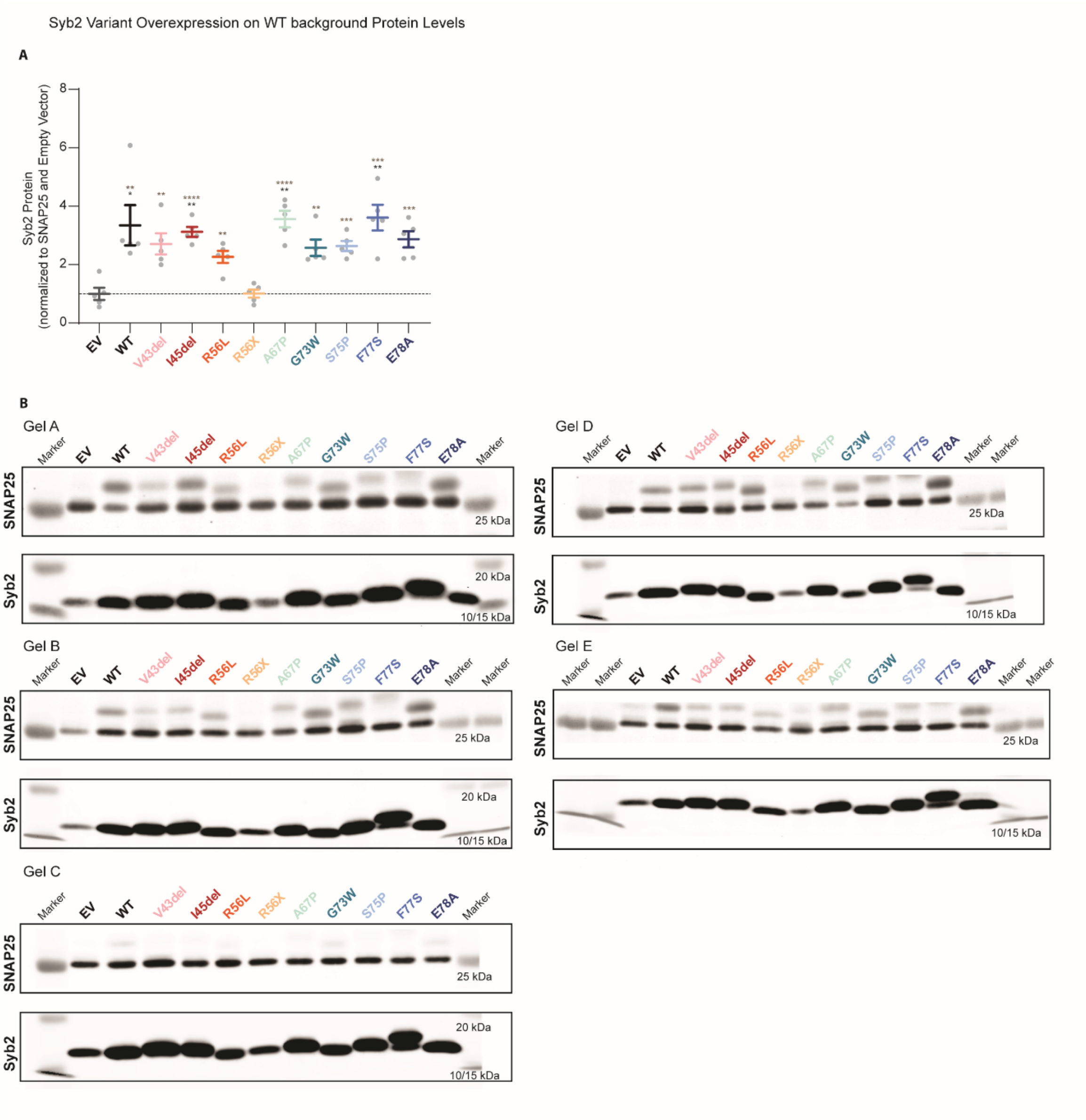

Supplementary Figure 1. (A) Quantification of Syb2 protein levels in empty vector control, WT, and Syb2 patient variant overexpression on a WT background. All protein levels normalized to SNAP25 expression and empty vector control. **(B)** Western blot images presented in gray scale displaying bands of interest of SNAP25 (control) and Syb2. The truncation mutation, R56X, is not recognized by the Syb2 antibody (per manufacturer: Botox B Syb2 cleavage product (AA 1 – 76) not recognized due to conformational masking). Values are mean ± SEM. Significance reported as *p < 0.05, **p < 0.01, ***p < 0.001, and ****p < 0.0001 in black for multiple comparison analysis and in brown for pairwise comparisons between each variant and empty vector. Exact p-values, n numbers, and additional statistical information provided in Dataset S2.

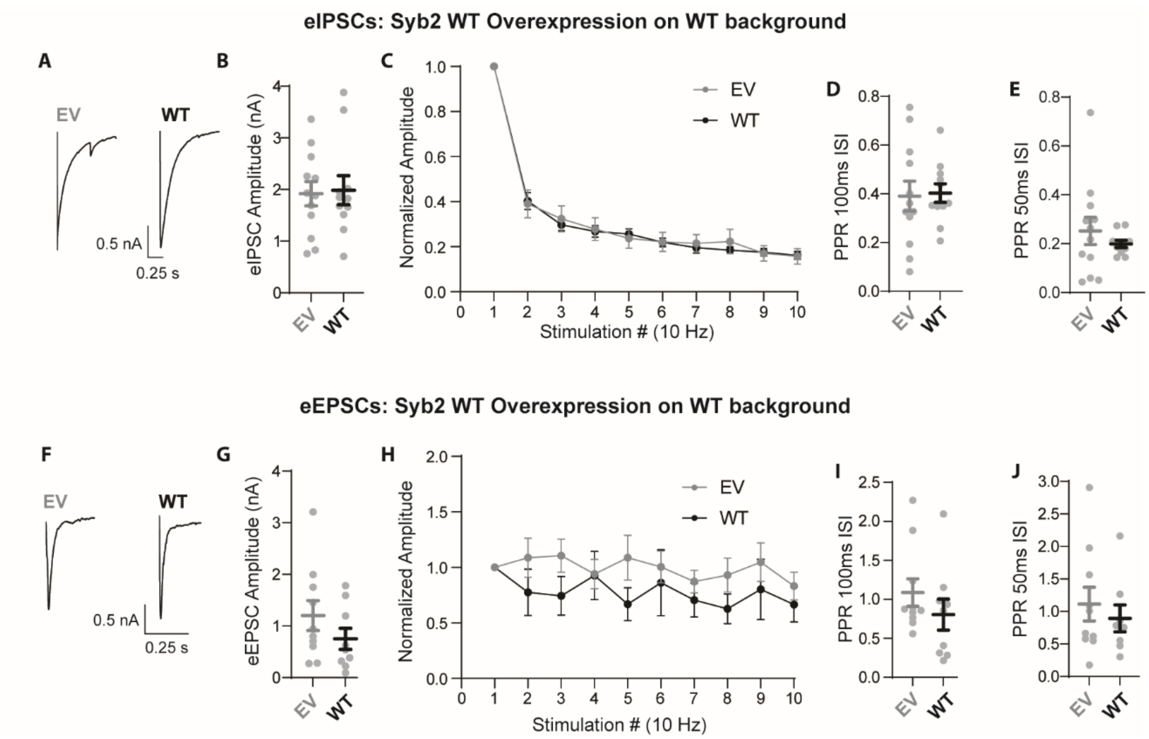

Supplementary Figure 2. **(A)** eIPSC representative traces of empty vector control and WT Syb2 overexpressed in a WT genetic background. **(B)** Quantification of eIPSC amplitude. **(C)** Normalized amplitude in response to repetitive stimulation (10Hz), **(D)** with the first two responses used to calculate paired pulse ratio at 10 Hz and **(E)** 20Hz. **(F)** eEPSC representative traces of empty vector control and WT Syb2 overexpressed in a WT genetic background. **(G)** Quantification of eEPSC amplitude. **(H)** Normalized amplitude in response to repetitive stimulation (10Hz), **(I)** with the first two responses used to calculate paired pulse ratio at 10 Hz and **(J)** 20Hz. WT data used in Figure 2 and SFigure3. Values are mean ± SEM. Significance reported as *p < 0.05, **p < 0.01, ***p < 0.001, and ****p < 0.0001. Exact p-values, n numbers, and additional statistical information provided in Dataset S2.

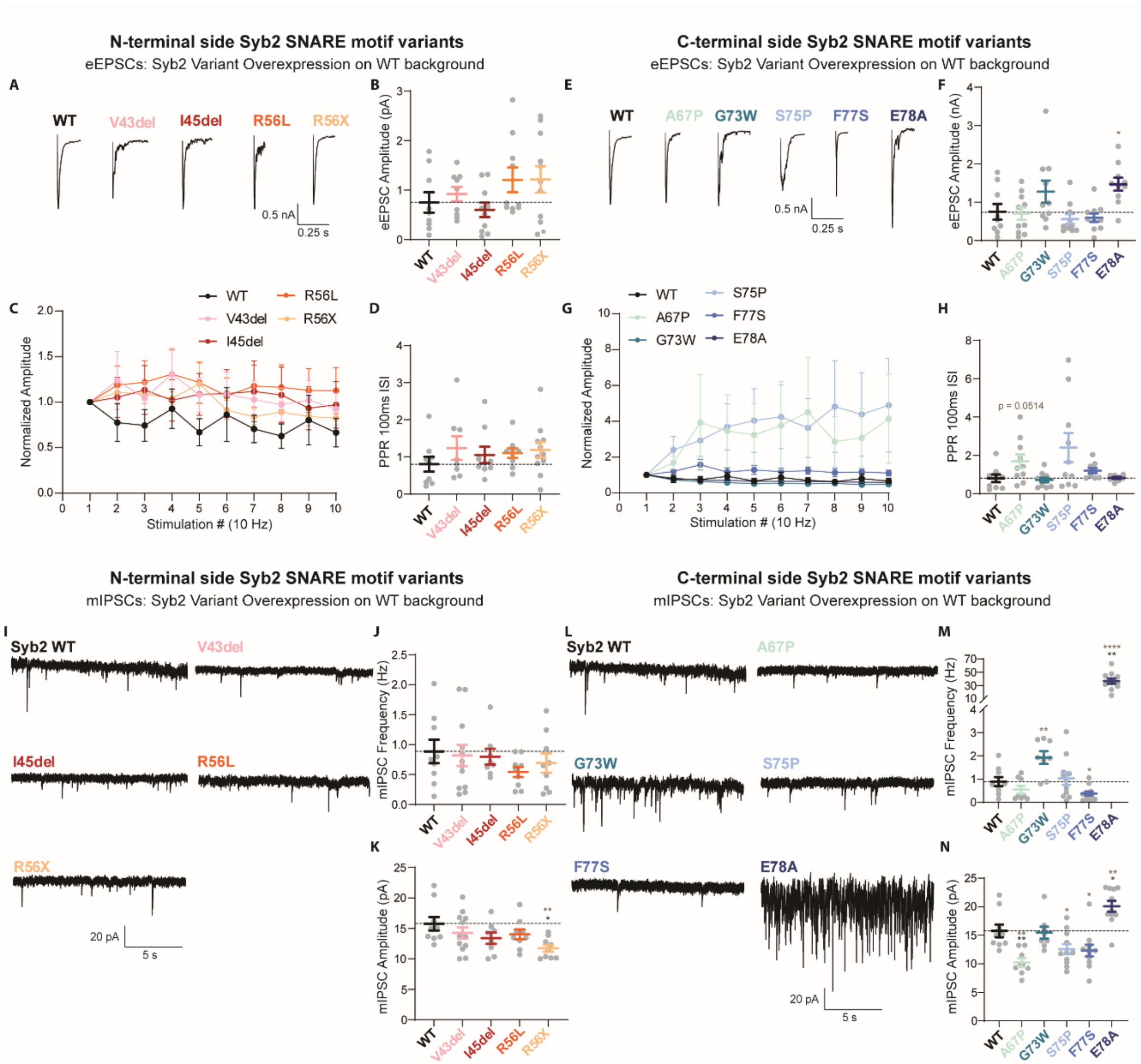

Supplementary Figure 3. (A) Representative traces of WT Syb2 and N-terminal side Syb2 SNARE motif variants (including layer 0 variants) overexpressed in a WT genetic background. **(B)** Quantification of eEPSC amplitude (average of 10 stim at 0.1 Hz) and **(C)** repetitive stimulation (10Hz), **(D)** with the first two responses used to calculate paired pulse ratio. **(E)** Representative traces of WT Syb2 and C-terminal side Syb2 SNARE motif variants overexpressed in a WT genetic background. (**F**) Quantification of eEPSC amplitude in response to one stimulation and **G,** repetitive stimulation (10Hz), **(H)** with the first two responses used to calculate paired pulse ratio. **(I)** mIPSC example traces of WT Syb2 and N-terminal side Syb2 SNARE motif variants (including layer 0 variants) overexpressed in a WT genetic background. **(J)** Quantification of mIPSC frequency and **(K)** amplitude. **(L)** mIPSC example traces of WT Syb2 and C-terminal side Syb2 SNARE motif variants overexpressed in a WT genetic background. **(M)** Quantification of mIPSC frequency and **(N)** amplitude. WT data is the same for N-terminal and C-terminal graphs. Values are mean ± SEM. Significance reported as *p < 0.05, **p < 0.01, ***p < 0.001, and ****p < 0.0001 in black for multiple comparisons analysis and in brown for pairwise comparisons between each variant and WT. Exact p-values, n numbers, and additional statistical information provided in Dataset S2.

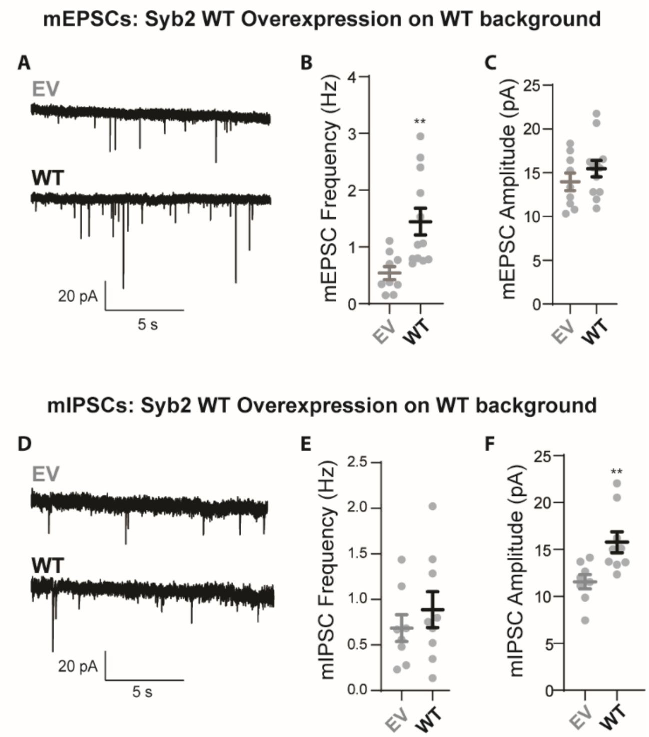

Supplementary Figure 4. (A) mEPSC example traces of empty vector control and WT Syb2 overexpressed in a WT genetic background. **(B)** Quantification of mEPSC frequency and **(C)** amplitude. **(D)** mIPSC example traces of empty vector control and WT Syb2 overexpressed in a WT genetic background. **(E)** Quantification of mIPSC frequency and **(F)** amplitude. WT data used in Figure 2 and SFigure3. Values are mean ± SEM. Significance reported as *p < 0.05, **p < 0.01, ***p < 0.001, and ****p < 0.0001. Exact p-values, n numbers, and additional statistical information provided in Dataset S2.

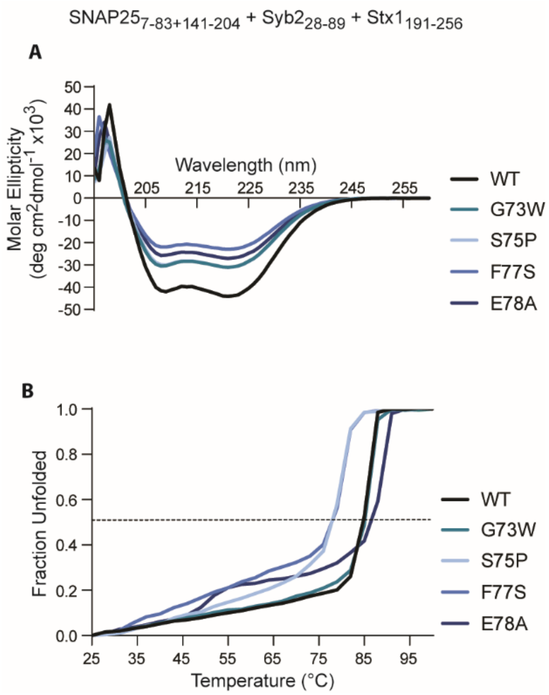

Supplementary Figure 5. (A) Circular dichroism spectra of SNARE complexes with SNAP25A$_{7\text{-}83,141\text{-}204}$, Syb2$_{28\text{-}89}$, Syntaxin1A$_{191\text{-}256}$ of WT, G73W, S75P, F77S, and E78A Syb2. **(B)** Thermal melting curves of SNARE complexes observed at 220 nm. Same data for WT, G73W, and E78A used in Figure 5. Values are mean ± SEM, except panel B is only mean.

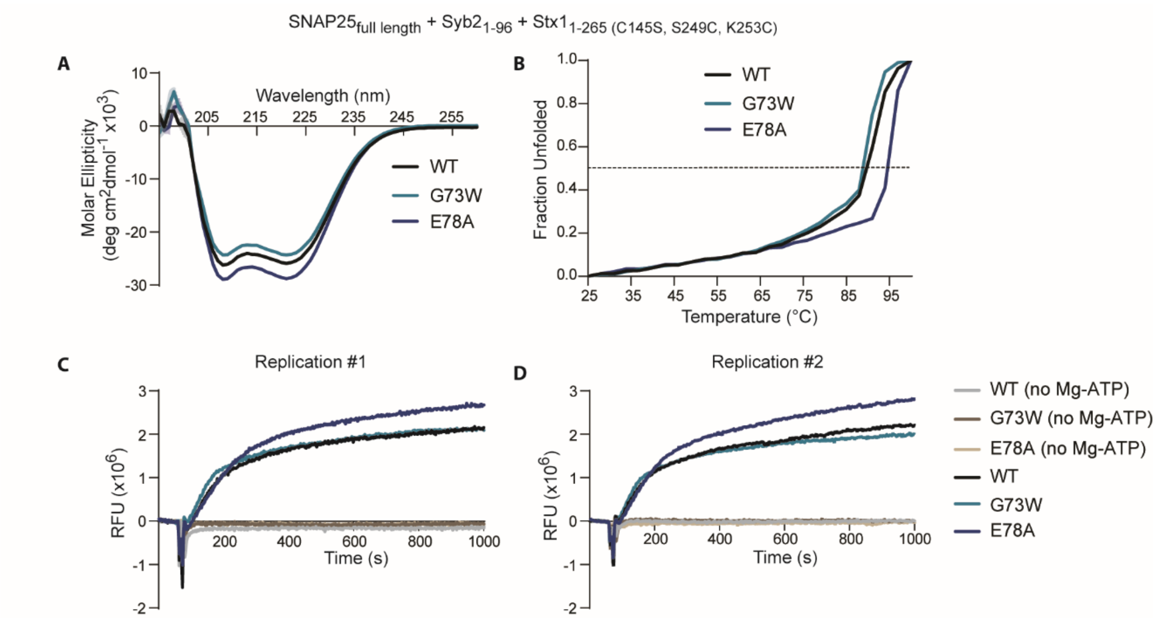

Supplementary Figure 6. (A) Circular dichroism spectra of SNARE complexes with SNAP25A$_{1\text{-}209}$, Synaptobrevin2$_{1\text{-}96}$, Syntaxin1A$_{1\text{-}265\ (C145S,\ S249C,\ K253C)}$ of WT, G73W, and E78A Syb2. **(B)** Thermal melting curves of SNARE complexes observed at 220 nm. **(C-D)** Replication of experiments in Figure 5D-E of NSF-mediated disassembly kinetics of the SNARE complex with WT Syb2, G73W Syb2, E78A Syb2 with and without Mg-ATP to initiate NSF mediated activity. Of note, the labeling efficiency was higher in the replication experiments using new preparations.

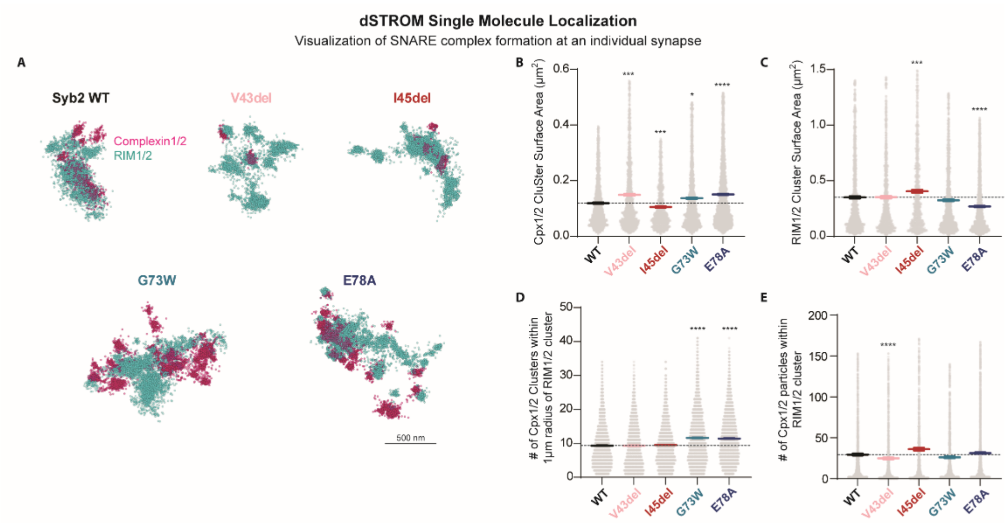

Supplementary Figure 7. (A) dSTORM single molecules localizations of identified pre-synaptic clusters using DBSCAN and proximity based analysis following WT Syb2 or patient variant Syb2 overexpression on a WT background. **(B)** Of defined synapses, complexin cluster and **(C)** RIM1/2 cluster surface area were quantified. **(D)** The number of complexin clusters within a 1 μm radius of a RIM cluster. **(E)** Individual complexin single molecules localized within the α-hull of a RIM cluster. Same data for WT, G73W, and E78A used in Figure 6. Values are mean ± SEM. Significance reported as *$p < 0.05$, **$p < 0.01$, ***$p < 0.001$, and ****$p < 0.0001$. Exact p-values, n numbers, and additional statistical information provided in Dataset S2.
